# Beat encoding at mistuned octaves within single electrosensory neurons

**DOI:** 10.1016/j.isci.2023.106840

**Published:** 2023-05-13

**Authors:** Alexandra Barayeu, Ramona Schäfer, Jan Grewe, Jan Benda

**Affiliations:** 1 Neuroethology, Institute for Neurobiology, Eberhard Karls University, 72076 Tübingen, Germany; 2 Bernstein Center for Computational Neuroscience Tübingen, 72076 Tübingen, Germany; 3 Werner Reichardt Centre for Integrative Neuroscience, 72076 Tübingen, Germany

**Keywords:** Behavioral neuroscience, Animal science, Modeling signal processing system

## Abstract

Beats are slow periodic amplitude modulations resulting from the superposition of two spectrally close periodic signals. The difference frequency between the signals sets the frequency of the beat. A field study in the electric fish *Apteronotus rostratus* showed the behavioral relevance of very high difference frequencies. Contrary to expectations from previous studies, our electrophysiological data show strong responses of p-type electroreceptor afferents whenever the difference frequency approaches integer multiples (mistuned octaves) of the fish’s own electric field frequency (carrier). Mathematical reasoning and simulations show that common approaches to extract amplitude modulations, such as Hilbert transform or half-wave rectification, are not sufficient to explain the responses at carrier octaves. Instead, half-wave rectification needs to be smoothed out, for example by a cubic function. Because electroreceptive afferents share many properties with auditory nerve fibers, these mechanisms may underly the human perception of beats at mistuned octaves as described by Ohm and Helmholtz.

## Highlights

• Electrosensory neurons respond to slow beats at high difference frequencies
• Extraction of aliased beat frequencies by smoothed out half-wave rectification
• Hypothesis: Human beat perception at mistuned octaves originates in hair cell synapse

## Graphical abstract

**Figure:**
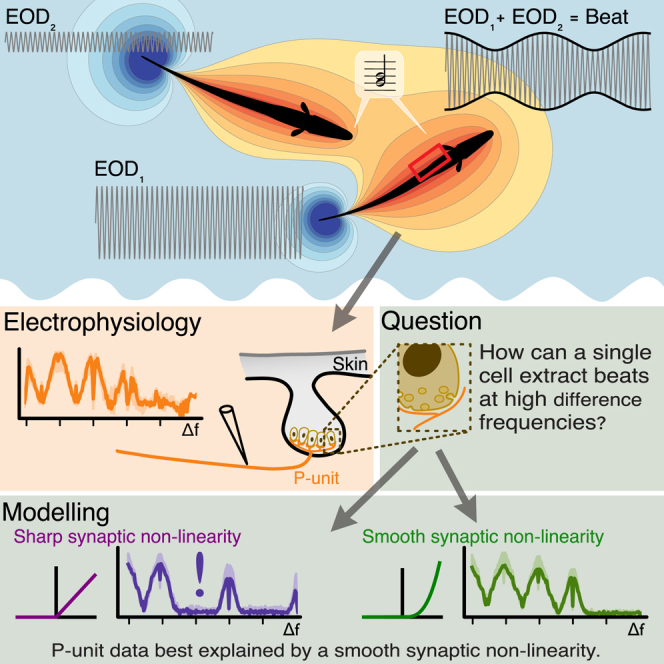

## Introduction

Periodic signals are key stimuli for the auditory system^1^^,^^2^^,^^3^ and the electrosensory system of wave-type electric fish.^4^ The superposition of two periodic signals with similar frequencies results in a periodic amplitude modulation (AM) known as “beat”. The frequency of such a beat is given by the frequency difference between the two signals and the beat amplitude is the one of the smaller of the two signals. Auditory beats give rise to a unique beating perception.^5^^,^^6^ In wave-type electric fish, beats play a central role in electrocommunication.^7^

Wave-type gymnotiform electric fish generate a sinusoidal electric organ discharge (EOD) of a species and individual specific frequency.^8^^,^^9^ The EODs of two nearby fish superimpose and thus produce a beat. Beat amplitude declines with the distance between the two fish and thus is strongly influenced by relative movement.^10^ The periodic beat is modulated by various types of electrocommunication signals on time scales ranging from 10 m to many seconds.^11^^,^^12^ Cutaneous tuberous organs that are distributed all over the body^13^ sense the actively generated electric field and its modulations. Within a single tuberous electroreceptor organ about 30 primary electroreceptors form ribbon synapses onto the dendrites of the same afferent fiber^14^^,^^15^ which projects via the lateral line nerve to the hindbrain. There it synapses onto pyramidal neurons in the electrosensory lateral line lobe.^16^ So far, the locus of action potential generation is not known, but most likely right after where the dendrites merge into a single fiber.

The time course of the activity of the p-type electroreceptor afferents (P-units) follows the time course of AMs of the EOD,^17^^,^^18^^,^^19^ similar to auditory fibers.^20^ So far, P-unit tuning to beat frequencies has been analyzed in a range up to 300 Hz in *Apteronotus leptorhynchus*.^21^^,^^22^^,^^23^ Beyond the strongest firing rate modulations in response to beat frequencies of 60–100 Hz the response declines down to baseline at about 250 Hz, with no firing rate modulations expected at even higher beat frequencies. Recent field studies on *Apteronotus*, however, demonstrated behaviorally relevant difference frequencies beyond 300 Hz in the context of courtship and synchronization of spawning^24^ and potential inter-species detection.^9^

Here we study how difference frequencies greater than 300 Hz are encoded in the electrosensory system. We recorded P-unit activities of *Apteronotus leptorhynchus* in response to a much wider range of difference frequencies (−750≤Δf≤2500 Hz) than before. By mathematical reasoning and simulations of integrate-and-fire neurons we identify the minimum functional requirements on single sensory cells to be able to extract and respond to slow envelopes that we find to be generated at large difference frequencies. Finally, we test our insights in behavioral experiments based on the jamming avoidance response (JAR,^25^).

Our results generate an interesting hypothesis for the mechanism underlying the perception of beats at mistuned octaves in humans. Experiments dating back to the 19th century demonstrated a perception of beats not only for spectrally close frequencies but also for mistuned octaves where the second frequency is close to octaves of the carrier frequency.^5^^,^^26^^,^^27^^,^^28^ The physiological mechanisms underlying these percepts remain an open issue.^6^^,^^20^

## Results

We recorded the spiking activity of n=40 p-type electroreceptors afferents (P-units) of the electric fish *A. leptorhynchus* in response to sinusoidal electrical stimuli with absolute frequencies ranging from 20 up to 3200 Hz. These stimuli superimpose with the fish’s own electric field and result in beat-like envelopes. In the following we speak of “beats” for difference frequencies between the stimulus frequency and the EOD frequency of the fish below half an octave. For larger difference frequencies we will use the term “beat-like envelopes”.

### Responses to low difference frequencies

**Figure 1:**
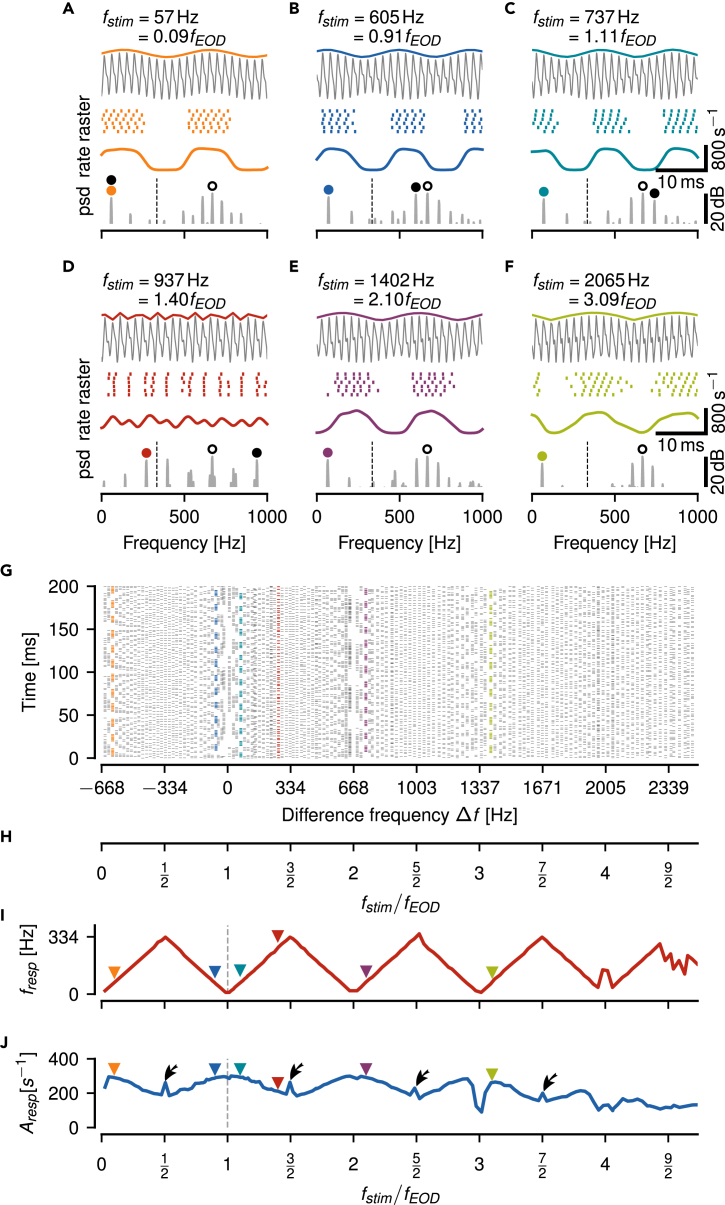
Encoding of a wide range of difference frequencies in an example P-unit (A–F) Beating envelopes (colored lines) of the carrier EOD superimposed with a sine wave stimulus (gray, top), spike raster and firing rate of evoked P-unit responses (center), and corresponding power spectrum (psd, bottom, open circle: $f_{EOD}$, dashed line: $f_{EOD}/2$, colored circle: strongest frequency below $f_{EOD}/2$, black circle: $f_{\mathrm{stim}}$) of a few selected stimulus frequencies as indicated. See Video S1 for cell responses to all stimulus frequencies. (G) Vertical raster plots showing spiking responses to a wide range of stimulus frequencies (one trial per frequency) indicate a repetitive structure of P-unit responses. Colored rasters mark examples shown in panels A–F. (H) Alternative stimulus frequency axis in multiples of $f_{EOD}$, i.e. $\Delta f/f_{EOD}+1$. (I) Frequency tuning, $f_{resp}$, of the P-unit response, i.e. frequency of its firing rate modulation, retrieved as the strongest peak in the response spectrum below $f_{EOD}/2$, repeats every integer multiple of $f_{EOD}$. Colored triangles mark examples shown in panels A–F. (J) Amplitude tuning curve, quantified as the amplitude of the peak at $f_{resp}$ (square-root of the integral below the peak at $f_{resp}$ of the power spectrum of the spike response convolved with a Gaussian kernel with σ=0.5 ms), also repeats at harmonics of $f_{EOD}$. Strongest responses are close to multiples of $f_{EOD}$. Exactly at odd multiples of $f_{EOD}/2$ peak amplitudes are increased (arrows).

P-units are known to respond to low-frequency beats by modulating their firing rate.^18^^,^^19^^,^^21^^,^^23^ For a fish with an EOD frequency $f_{EOD}=668$ Hz and a stimulus with frequency $f_{stim}=737$ Hz, mimicking a fish with higher EOD frequency, we get a beat at the difference frequency $\Delta f=f_{stim}-f_{EOD}=69$ Hz (Figure 1C, top). A P-unit responds to this AM as demonstrated by the spike raster and the corresponding time-resolved firing rate (Figure 1C, center). The strongest peaks in the power spectrum of the spike response are at the beat frequency (colored marker) and the receiver’s EOD frequency (gray circle, Figure 1C bottom).

When $f_{stim}$ is below $f_{EOD}$ (here Δf=−63 Hz), the resulting beat is similar to the one discussed above, although at a negative difference frequency. The P-unit response has the same features as for the positive difference frequency and the spectrum has prominent peaks at the same locations (compare Figures 1B  and 1C). Signals generated by positive or negative Δf differ only in small phase shifts in the carrier, encoded by another population of electroreceptors, the T-units.^29^

As $f_{stim}$ increases, the beat frequency increases accordingly and the strength of the P-unit’s response, characterized by the modulation depth of the P-unit’s firing rate, declines (Figure 1D), confirming previous results.^18^^,^^21^^,^^23^^,^^30^

### Envelope frequency does not match difference frequency for high stimulus frequencies

In the examples discussed so far, |Δf| and the frequency of the induced beating envelope are identical. Increasing (or decreasing) $f_{stim}$ beyond $f_{EOD}\pm f_{EOD}/2$, however, breaks this relation. Instead, at $f_{stim}=0.1f_{EOD}$, $2.1f_{EOD}$, or $3.1f_{EOD}$ the resulting beating envelopes have the same frequency as for $f_{stim}=1.1f_{EOD}$, the classical beat for a stimulus frequency close to the receiver’s EOD frequency (compare Figures 1A, 1C, 1E, and 1F). As the stimulus frequency gets close to integer multiples of the EOD frequency, the envelope frequency goes to zero even if the absolute difference frequency is larger than half the EOD frequency.

### P-units respond to an extremely wide range of stimulus frequencies

Slow envelopes of the fish’s EOD effectively modulate the P-unit’s spike responses. This is well known for |Δf| less than $f_{EOD}$/2. Beyond such frequencies we observe reoccurring ranges of stimulus frequencies that lead to strongly and slowly modulated responses up to stimulus frequencies of approximately three times the EOD frequency (Figure 1G and Video S1). Also, toward stimulus frequencies down to about 35 Hz, we observed clear P-unit responses (Figures 1A and 1G and first frames of Video S1). Stimulus frequencies leading to clear modulations of a P-unit’s firing rate are centered on integer multiples of $f_{EOD}$. Thus, $f_{EOD}$ defines the frequency scale in which $f_{stim}$ has to be interpreted. Accordingly, from now on we express $f_{stim}$ relative to $f_{EOD}$ $\left(f_{stim}/f_{EOD}\right)$, which also allows for comparisons across animals with distinct EOD frequencies (Figure 1H).

Video S1. Animation of P-unit responses to the full range of recorded difference frequencies, related to and same cell as in Figure 1 A Vertical raster plots with one trial per stimulus frequency, B frequency tuning, and C amplitude tuning of the cell. Same as in Figures 1G, 1I, and 1J. Orange raster and triangles mark stimulus frequency shown in D. D Beating envelopes (orange) of the carrier EOD superimposed with a sine wave stimulus (gray, top), spike raster and firing rate of evoked P-unit response (center), and corresponding power spectrum (psd, bottom, open circle: $f_{EOD}$, dashed line: $f_{EOD}/2$, orange circle: strongest frequency below $f_{EOD}/2$) for all stimulus frequencies recorded in this cell.

### Aliasing structure of beat responses

Around integer multiples of $f_{EOD}$ we see slow beat-like signals and correspondingly strongly modulated spike responses. Toward odd multiples of $f_{EOD}/2$, signal envelopes get faster and spike responses get weaker. We quantified P-unit response characteristics by extracting the frequency and the respective strength of the response modulation from the power spectrum of the spiking response. The position of the strongest peak in the response power spectrum below $f_{EOD}/2$ is the frequency of the P-unit’s firing modulation. Plotting this frequency as a function of $f_{stim}$ reveals a repetitive pattern shaped like a “Toblerone” (Figure 1I). This zigzag pattern is reminiscent of aliasing known from the sampling theorem, with $f_{EOD}/2$ playing the role of the Nyquist frequency.

For stimulus frequencies evoking no or only small peaks in a P-unit’s response spectrum, the spectrum was dominated by the P-unit’s baseline spectrum with a peak at or close to the baseline firing rate. When the amplitude of this baseline peak was larger than the amplitude of the low-frequency alias of the stimulus, then the frequency tuning curve deviated from the zigzag pattern. This may happen around stimulus frequencies close to multiples of $f_{EOD}$ (Figure 1I at 2005 Hz) or when the stimulus frequency exceeded the P-unit’s tuning range (Figures 3B and 3D).

### Periodic amplitude tuning curve

The amplitude $A_{resp}$ of the respective peak in the response spectrum reflects the strength of the P-unit response, i.e. the modulation depth of its time-resolved firing rate. The resulting tuning curve also shows a repetitive structure (Figure 1J). Close to multiples of $f_{EOD}$ the response is strongest. These maxima, however, become smaller the higher $f_{stim}$. Directly at the multiples we observe dips in the response amplitudes which can be attributed to the P-unit’s spike-frequency adaptation.^19^ Response amplitudes decline as the stimulus frequency approaches odd multiples of $f_{EOD}/2$. Exactly at odd multiples of $f_{EOD}/2$, response amplitudes are often markedly elevated (arrows in Figure 1J), because here two peaks in the response spectrum cross each other (Video S1). For higher stimulus frequencies response amplitude increases again toward the next multiple of $f_{EOD}$. This increase of the response amplitude beyond $f_{stim}=\frac{3}{2}f_{EOD}$ was not expected given previous data on the encoding of AMs in P-units.^18^^,^^21^^,^^23^^,^^30^

### Amplitude tuning depends on post-synaptic filtering

**Figure 2:**
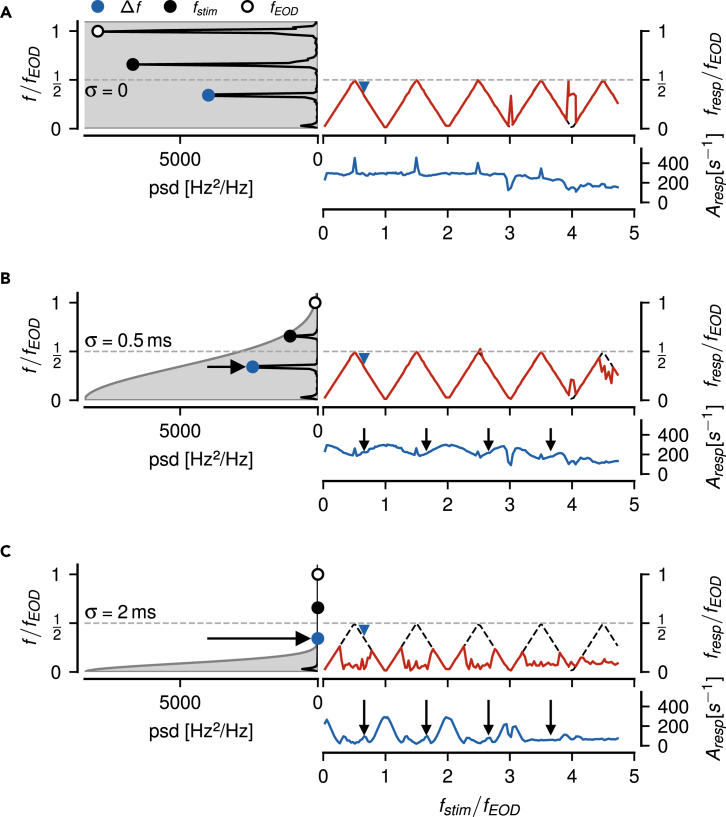
Influence of post-synaptic low-pass filtering on P-unit responses to beat-like stimuli Left: Power spectral density of a P-unit’s firing rate response (unit of firing rate squared, ${\text{Hz}}^{2}$, per frequency bin, ${\text{Hz}}^{-1}$, here not transformed to decibel) to a Δf=−220 Hz beat (blue markers). The convolution kernel mimicking post-synaptic filtering is indicated by the Gaussian-shaped gray area. The horizontal dashed-dotted line indicates $f_{EOD}/2$. Right: Frequency tuning curve (top) together with the expected aliased frequencies (black dashed line) and amplitude tuning curve (bottom) estimated from the strongest peak of a P-unit’s response below $f_{EOD}/2$. (A) Spectrum and tuning curves of the raw, binary spike trains recorded with a resolution of 40 kHz. The highest peak in the spectrum is at $f_{EOD}$ (open circle), followed by the peak at the absolute value of the stimulus frequency $f_{stim}$ (black circle). The peak at the difference frequency corresponding to the frequency of the resulting beat is even smaller, but is the largest peak below $f_{EOD}/2$ (orange circle). Frequency tuning follows the aliased frequencies over almost the whole measured range up to $5f_{EOD}$. The amplitude tuning curve is mostly flat with pronounced peaks at odd multiples of $f_{EOD}/2$. (B) A biological plausible post-synaptic filter, modeled by convolving the spike trains with a Gaussian kernel (σ=0.5 ms), keeps the frequency tuning, but reduces the amplitude of the P-unit’s response for stimulus frequencies close to odd multiples of $f_{EOD}/2$ (arrows). (C) A wider post-synaptic potential, modeled by a Gaussian with σ=2 ms, degrades the frequency tuning curves and strongly modulates amplitude tuning.

The synapse between P-unit electroreceptor afferents and their target neurons in the hindbrain, pyramidal neurons in the electrosensory lateral line lobe, introduces a fast excitatory postsynaptic potential of about 1 ms duration.^31^ Postsynaptic potentials are the physiological equivalent of bins or filter kernels used to compute time-resolved firing rates, therefore our estimation of P-unit firing rates — as any method used for estimating firing rates — already includes assumptions about the postsynaptic readout. The shape of the P-unit’s amplitude tuning curve strongly depends on the width of the chosen filter kernel, because it low-pass filters the spike train, as does a postsynaptic potential. P-unit tuning is relatively flat when computed directly from the spike trains (no low-pass filtering, Figure 2A), but becomes more modulated the more the spike train is low-pass filtered, because the wider the postsynaptic potential, the more the response peaks closer to $f_{EOD}/2$ are attenuated (arrows in Figure 2B and 2C). Low-pass filtering the spike train with a physiologically plausible Gaussian kernel with σ=0.5 ms attenuates responses to high envelope frequencies while leaving responses to low envelope frequencies untouched, resulting in a periodically modulated amplitude tuning curve (Figure 2B).

### Sensitive cells respond to a larger frequency range

P-unit responses scale with stimulus amplitude^17^^,^^18^ and different P-units differ in their sensitivities to a global stimulus.^32^ To account for both, sensitivity and stimulus intensity, we quantified the P-unit’s response amplitude to a standard stimulus, a beat at Δf=50 Hz.^32^ Across all our recorded P-units, firing rate modulations evoked by the 50 Hz beat ranged from 70 to 360 Hz and were positively correlated with baseline firing rates ranging from 80 to 527 Hz (r=0.63, p<0.0001). Think of the modulation depth as the effective stimulus amplitude driving the P-unit’s response.

**Figure 3:**
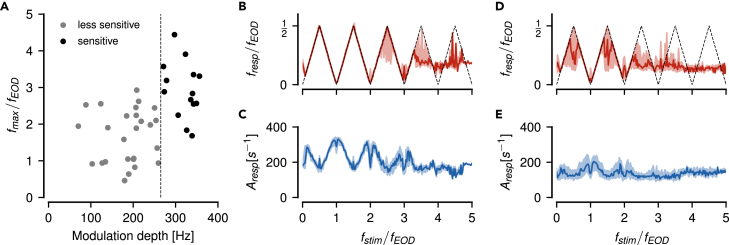
Dependence of the aliasing structure on sensitivity (A) For each of the recorded P-units we estimated the maximum stimulus frequency $f_{\max}$ up to which its frequency tuning curve indicated a response by following the predicted low-frequency aliases of the stimulus frequency (e.g. dashed line in panel B). The more sensitive a P-unit, as quantified by the modulation depth of its firing rate response to a 50 Hz standard beat, the higher the maximum frequency. Because we were primarily interested in understanding the mechanisms behind the aliasing structure of the P-units’ responses, we focused our analysis on the most sensitive cells with modulation depths greater than 265 Hz (black). (B) Frequency tuning of the n=14 most sensitive cells reaches up to almost four multiples of $f_{EOD}$ (median with interquartile range). (C) Corresponding amplitude tuning curves are of course stronger in comparison to the less sensitive cells. (D and E) Frequency and amplitude tuning curves of the n=26 less sensitive cells (gray dots in A).

The stronger the modulation depth of a P-unit’s response, the more the P-unit tuning curves follow the low-frequency alias of the stimulus to higher stimulus frequencies (Pearson’s r=0.51, p=0.001, Figure 3A). Because we were primarily interested in understanding the mechanisms behind the aliasing structure of the P-units’ responses, we focused our analysis on the n=14 most sensitive cells with modulation depths greater than 265 Hz. These cells respond up to almost four times $f_{EOD}$ (Figures 3B and 3C) whereas the less sensitive cells responded on average just up to about twice $f_{EOD}$ (Figures 3D and 3E).

### Algorithms for extracting envelope frequencies at high stimulus frequencies

In the following sections we explore prerequisites necessary for neurons to extract the low-frequency alias of the stimulus making up the AMs of beats. The resulting theory fully explains the experimental observations, including the enhanced responses at odd multiples of $f_{EOD}/2$ (arrows in Figure 1J). The details of the mathematical derivations and equations are provided in Data S1.

### Slow beating envelopes in superimposed cosine waves

As suggested by Figure 1, the aliasing structure of the P-unit response results from the envelopes of the interacting EODs. To understand how such responses can arise, we need to understand the mechanism by which envelopes are retrieved from the superposition of the two EODs. Transformed to the Fourier domain, we ask how a spectral peak at the envelope frequency can be generated.

This is a generic problem independent of the electrosensory system, and thus we express the problem in terms of two cosine waves: a carrier, the EOD of the receiving fish, with frequency $\omega_{1}=2\pi f_{1}$ and amplitude one and a stimulus, the EOD of another fish, with frequency $\omega_{2}=2\pi f_{2}$ and an amplitude α measured relative to the amplitude of the carrier (stimulus contrast). As we point out below, we mainly focus on the case α≪1, where we have a clear distinction between a large amplitude carrier signal with frequency $\omega_{1}$ and a stimulus of smaller amplitude at frequency $\omega_{2}$.

Both signals superimpose:

(Equation 1) $$x\left(t\right)=\cos\left(\omega_{1}t\right)+\alpha \;\cos\left(\omega_{2}t\right)$$

**Figure 4:**
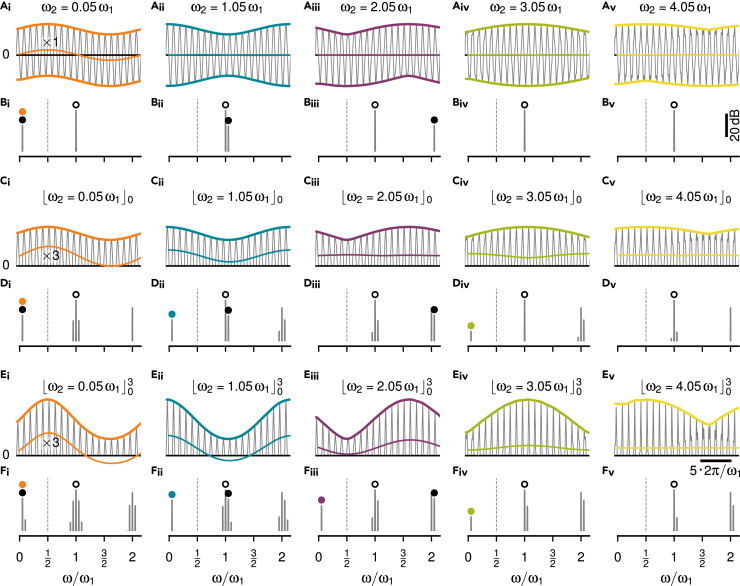
Slow envelopes resulting from the superposition of two cosine waveforms (A) The raw signals — superpositions of a cosine carrier with fixed frequency $\omega_{1}$ and stimulus cosines of various frequencies $\omega_{2}$ close to multiples of $\omega_{1}$ as indicated and an amplitude of α=0.2 relative to the one of the carrier. Upper and lower envelopes are highlighted by thick colored lines. The thin colored lines are running averages over a single cycle of the carrier frequency, which keep only frequency components smaller than $\omega_{1}$. For stimulus frequencies similar to or larger than the carrier frequency, the running average is zero, even when the upper and lower envelopes are out of phase. (B) Although slow envelopes are clearly visible in the waveforms shown in A, the corresponding power spectra have only peaks at $\omega_{1}$ (open circle) and $\omega_{2}$ (black circle) and not at the slow envelopes. Only when the stimulus frequency $\omega_{2}$ is below $\omega_{1}/2$ it coincides with the envelope (orange circle in $B_{i}$). (C) Thresholding cuts away the negative half-waves and leaves only the upper envelope intact. Now the running average (enlarged by a factor of three around its mean) follows the envelopes for $\omega_{2}$ close to 0, 1, and 3 multiples of $\omega_{1}$, but not for 2 and 4 multiples. Note, for $\omega_{2}=3.05\;\omega_{1}$ the running average is antiphasic to the envelope. (D) Thresholding generates frequencies explaining the envelopes at odd multiples of the carrier frequency only (cyan and green circles in $D_{ii}$ and $D_{iv}$). (E) Raising the thresholded signal from C to a power of three narrows the half-waves of the carrier and slightly distorts the envelopes. The running average (enlarged by a factor of three) follows the upper envelope up to three multiples of $\omega_{1}$, but with decreasing amplitudes. (F) This operation adds a peak in the power spectrum also for stimulus frequencies around the second multiple of the carrier (purple circle in $F_{iii}$).

The resulting signal x(t) also shows the characteristic beating envelopes reoccurring at multiples of the frequency $\omega_{1}$ of the carrier signal (Figures 4A_i_–A_v_). A running average that attenuates the fast carrier signal, however, results in flat lines, except for stimulus frequencies close to zero (compare Figures 4A_i_ and A_ii_–A_v_). This happens even for $\omega_{2}$ close to twice and four times $\omega_{1}$, where upper and lower envelopes are out of phase, because the stimulus cosine distorts the carrier cosine. The respective power spectra have peaks only at the original stimulus frequencies $\omega_{1}$ and $\omega_{2}$ (Figures 4B_i_–B_v_). There are no spectral peaks at the slow envelope frequencies (except for $\omega_{2}$ close to zero), although the envelopes are clearly visible.

### Neither the analytic signal nor squaring explains the aliasing structure of the beating envelopes

A non-linear operation needs to be applied to the signal to generate additional spectral peaks at the observed envelope frequencies. Commonly used non-linearities to retrieve the AM of a beat for two spectrally close signals are the absolute value of the analytic signal obtained by means of a Hilbert transformation, squaring, or thresholding.^33^^,^^34^

Both the analytic signal and squaring predict the frequency of the beating envelope to be identical to the difference frequency. This is exactly what we expect for low difference frequencies, i.e. for stimulus frequencies $\omega_{2}$ close to $\omega_{1}$. For higher difference frequencies, however, the analytic signal suggests an AM with growing frequency (Figure A1A in Data S1), although the signals are not necessarily amplitude modulated signals anymore (e.g., panels $A_{i}$, $A_{iii}$ and $A_{v}$ in Figure 4, upper and lower envelopes are no mirror versions of each other). Squaring also predicts a spectral peak at the difference frequency, no matter how large the difference frequency (Figure A1B in Data S1). These two types of non-linearities neither explain the aliasing structure we observe in superimposed cosines (Figure 4A), nor in superimposed EODs and the corresponding P-unit responses (Figure 1).

### Thresholding explains aliasing at odd multiples of the carrier frequency

A threshold non-linearity

(Equation 2) $${\left\lfloor x\left(t\right)\right\rfloor }_{0}=\left\{ \begin{array}{ccc} x\left(t\right) & ; & x\left(t\right)\geq 0\\ 0 & ; & x\left(t\right)<0 \end{array}\right.$$

sets all negative values of a signal to zero. Only the positive half-waves are passed through. The upper envelope is retained whereas the lower envelope of the signal is discarded (Figure 4C). For brevity we call this half-wave rectification “thresholding”. Thresholding indeed generates spectral peaks in the resulting signal at some of the envelope frequencies, but not for stimulus frequencies close to the second or fourth multiple of the carrier frequency (Figure 4D). Consequently, low-pass filtering the thresholded signals results in flat lines for $\omega_{2}$ close to two and four times of $\omega_{1}$, despite the obvious envelope. Surprisingly, the stimulus distorts the carrier such that for $\omega_{2}$ close to $2\omega_{1}$ the running average generates a signal with the same frequency as the envelope, but antiphasic to the envelope (Figure 4C_iv_).

**Figure 5:**
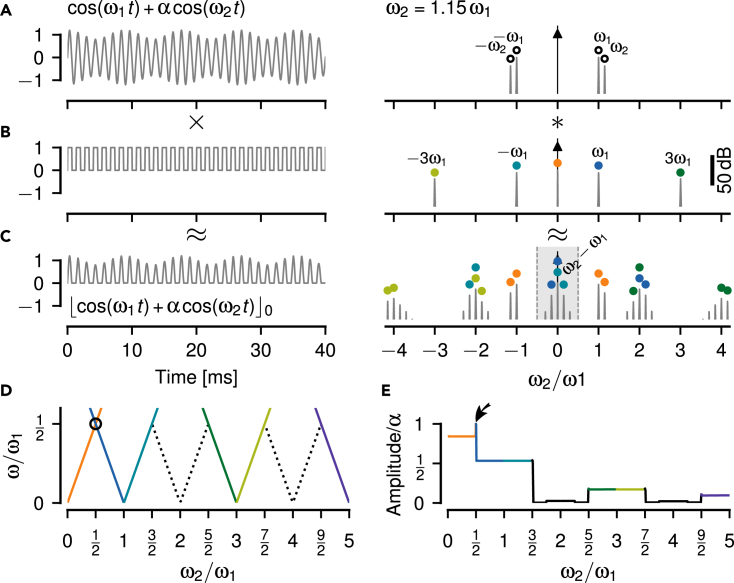
Thresholding is not sufficient to explain the full aliasing structure of beat-like envelopes (A) A beat generated by summing up two cosines with frequencies $\omega_{1}$ and $\omega_{2}$ (left) only contains these two frequencies in its Fourier spectrum (right). The amplitude α=20 % of the stimulus cosine is smaller than the one of the carrier. (B) A pulse train, Equation 4, used to approximate a threshold operation, Equation 2, has peaks at odd multiples of $\omega_{1}$ and at zero. (C) Thresholding the signal from A results in a rich spectrum. In the limit of vanishing stimulus amplitude α the thresholded signal can be approximated by multiplying the signal from A with the pulse train in B. The corresponding spectrum is approximated by convolving the spectrum of the signal with the one of the pulse train (colored circles, one color for each peak of the pulse train). Here, for $\omega_{2}=1.15\;\omega_{1}$ a peak appears at the difference frequency $\omega_{2}-\omega_{1}$ below $\omega_{1}/2$ (gray area). This peak describes the slow amplitude modulation of the beat visible in A. Because the stimulus amplitude is not close to zero, this approximation does not explain all the side peaks in the spectrum. (D) The position of peaks in the spectra of thresholded signals (colored lines) below $\omega_{1}/2$, as a function of stimulus frequency $\omega_{2}$. This curve is a prediction for the frequency tuning curves of P-units. The dashed line marks the expected alias frequencies of the stimulus, and the circle the only crossing of spectral peaks. Note, that close to two and four multiples of $\omega_{1}$ no peaks are created by thresholding below $\omega_{1}/2$. See Figure A1C in Data S1 for spectral peaks also at higher frequencies. (E) Amplitude of the peaks shown in D decrease with higher multiples of $\omega_{1}$. At the crossing of spectral peaks (circle in D) amplitudes sum up (arrow). These amplitudes are measured relative to the amplitude α of the stimulus cosine and would drive the amplitude tuning curves of P-units.

To make the threshold operation, Equation 2, analytically tractable, we approximate it by a multiplication of the signal (Figure 5A) with a pulse train of the same frequency $\omega_{1}$ as the carrier signal (Figure 5B):

(Equation 3) $${\left\lfloor x\left(t\right)\right\rfloor }_{0}\approx x\left(t\right)\cdot p\left(\omega_{1}t\right)$$

The pulse train

(Equation 4) $$p\left(\omega_{1}t\right)=\left\{ \begin{array}{ccc} 1 & ; & \cos\left(\omega_{1}t\right)\geq 0\\ 0 & ; & \cos\left(\omega_{1}t\right)<0 \end{array}\right.$$

multiplies positive half-waves of the carrier cosine with one and negative half-waves with zero (Figure 5C).

Note that this approximation is valid only in the limit α→0. For larger stimulus amplitudes the stimulus distorts the carrier, shifting also its zero crossings. As a consequence, additional side-peaks occur in the spectra (Figure 4D).

According to the convolution theorem a multiplication in time equals a convolution in the Fourier domain. Thus, the Fourier spectrum of the thresholded signal (Figure 5C) is given by the convolution of the spectrum of the signal (Figure 5A) and that of the pulse train (Figure 5B). The spectrum of the pulse train has a peak at zero frequency and peaks at all odd multiples of the carrier frequency $\omega_{1}$.

A first component of the resulting spectrum is the convolution of the carrier frequency, $\omega_{1}$, with the pulse train spectrum. This results in peaks at even multiples of $\omega_{1}$ and at $\pm \omega_{1}$ (horizontal lines in Figure A1C in Data S1). These frequency components do not make up the beating envelope, because they do not depend on $\omega_{2}$.

The second component of the spectrum, the convolution of the stimulus frequency $\omega_{2}$ with the pulse train, provides side peaks at $\pm \omega_{2}$ to all the peaks of the pulse train (Figure A1C in Data S1). These peaks explain the aliasing structure of the beating signal envelopes and thus the frequency tuning curves of P-units’ around odd multiples of $\omega_{1}$ and around zero frequency, but not around two times $\omega_{1}$ (Figure 5D). The amplitudes of these peaks quantify the amplitudes of the beating envelopes and drive the amplitude tuning curves of P-units. They decrease with higher multiples of the carrier (Figure 5E, see section 3 in Data S1 for a mathematical derivation of these amplitudes). Note that this decrease in amplitude is stronger than that of the signal’s envelopes.

In contrast to the amplitude of the analytic signal and to squaring, the threshold operation introduces many additional peaks in the spectrum. These are necessary for explaining some but not all of the aliasing structure of signal envelopes. In particular, thresholding does not generate low frequency spectral peaks for stimulus frequencies around twice the carrier frequency.

### Threshold cubed fills in frequencies at around twice the carrier frequency

How can we fill in the missing components in the spectrum around even multiples of $\omega_{1}$? The synaptic transfer function between P-units and pyramidal cells has not been measured yet, but P-units share several properties with inner hair cells in the auditory system and their transfer functions has been described by a power of three.^35^^,^^36^^,^^37^ Therefore, we first take the thresholded signal to a power of three:

(Equation 5) $$x_{c}\left(t\right)={\left\lfloor x\left(t\right)\right\rfloor }_{0}^{3}$$

In the resulting signals the half-waves of the carrier are narrower and the envelopes are slightly distorted in comparison to the pure threshold (Figure 4E). In the corresponding power spectra, we now get peaks at the slow envelope frequencies up to the third multiple of $f_{EOD}$ (Figure 4F). In particular, we get such a peak for stimulus frequencies close to the second multiple of $f_{EOD}$ (Figure 4F_iii_). Also the running average produces signals of the same frequency and phase as the envelopes up to the third multiple of $f_{EOD}$.

**Figure 6:**
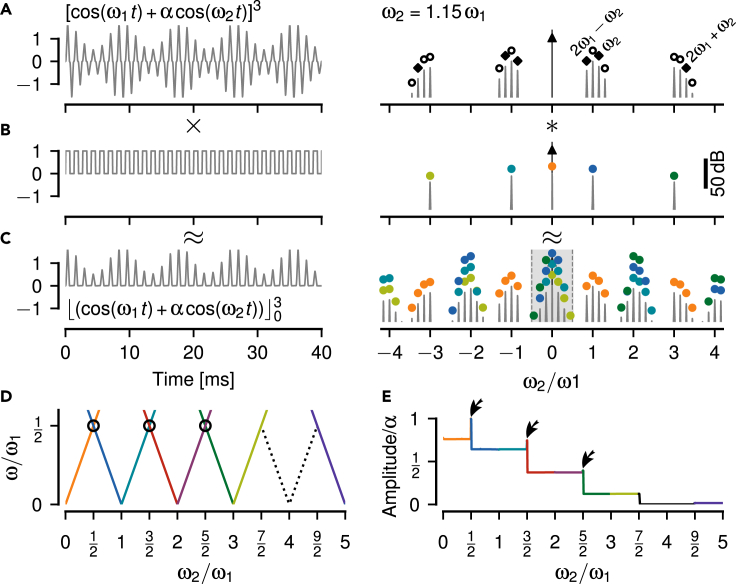
Cubing fills in frequencies corresponding to slow envelopes close to the second multiple of the carrier (A) The cubed signal (left) has a more pointed carrier waveform and eight peaks in the positive half of the Fourier spectrum (right, Figure A2A in Data S1). (B) Again we approximate the threshold operation by a multiplication with a pulse train. (C) The threshold-cubed signal has a much richer power spectrum compared to the thresholded signal without exponent (Figure 5C). The convolution process is illustrated by the colored markers — the spectrum of the cubed signal from A is shifted to each of the individually colored peaks in the spectrum of the pulse train from B. In this process many peaks fall on top of each other and add up. The gray shading marks frequencies below $\omega_{1}/2$. (D) Position of the largest spectral peaks of thresholded and cubed signals as a function of stimulus frequency. This is the frequency tuning curve. See Figure A2B in Data S1 for spectral peaks at higher frequencies. Dashed line indicates the expected alias frequencies of the stimulus, that are extracted by the cubed-threshold operation up to $\omega_{2}=3.5\;\omega_{1}$. Circles mark peak crossings. (E) Amplitudes of the peaks in D describe the amplitude of the beat-like signal envelopes. Crossing spectral peaks in D (circles) add up and result in elevated amplitudes (arrows). These amplitudes are driving the P-unit responses and thus are relevant for their amplitude tuning curve.

Again, this can be approximated by multiplying with a pulse train after taking the signal to the power of three (Figures 6A–6C). The spectrum of the two superimposed cosines cubed has $2^{3}=8$ peaks (two times convolution of two peaks with themselves, Figure A2A in Data S1). Of those the peaks at $\left|2\omega_{1}-\omega_{2}\right|=\left|\omega_{1}-\Delta \omega \right|$ are the only relevant additions in comparison to the threshold without exponent. The convolution of these peaks with the zero-frequency peak of the pulse train fills in the missing frequencies around twice the carrier frequency (Figure 6D, red and purple). This is enough for explaining the frequency tuning curve of P-units (Figure 3B) up to 3.5 multiples of $f_{EOD}$.

The amplitudes of the peaks below $\omega_{1}/2$ decline in a stepwise manner for each multiple of $\omega_{1}$ (Figure 6E). At $\omega_{1}/2$, $3\omega_{1}/2$, and $5\omega_{1}/2$ spectral peaks cross each other (circles in Figure 6D). Their respective amplitudes add up and result in elevated amplitudes exactly at these frequencies (Figure 6E, arrows). These peak crossings explain the elevated P-unit responses at these frequencies (arrows in Figure 1J). See section 4 in Data S1 for a mathematical derivation of peak frequencies and amplitudes and Figure A2B in Data S1. Again, the peak amplitudes decline much faster than the amplitudes of the envelopes.

In conclusion, a cubed threshold operation is sufficient to generate spectral peaks below half the carrier frequency for stimulus frequencies up to inclusively three multiples of $\omega_{1}$. These peaks correspond to the aliasing structure we observe in the envelopes of these signals and potentially explain the recorded P-unit responses. In contrast, a pure threshold fails to extract slow envelopes around twice the carrier frequency. In addition, the non-linear operation needs to be followed by a low-pass filter that isolates these envelope frequencies by attenuating the carrier frequency and other spectral peaks beyond $\omega_{1}/2$.

### Spiking dynamics cannot explain responses to higher beat frequencies

**Figure 7:**
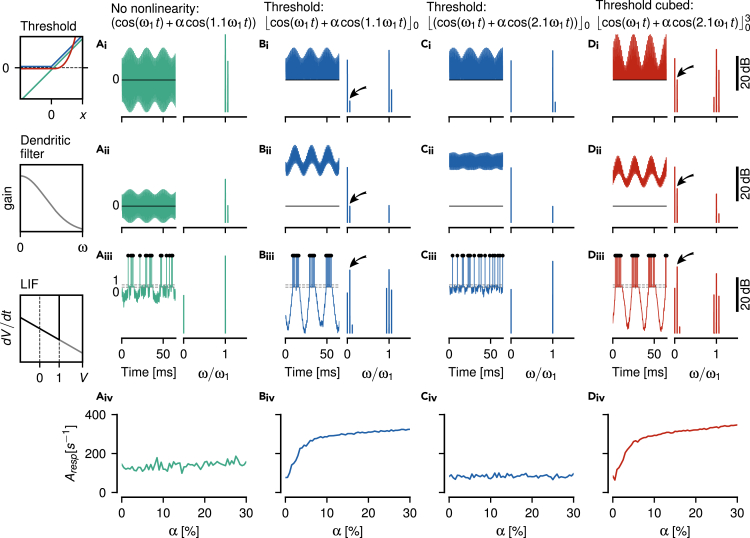
Integrate-and-fire models of P-unit spiking activity As an example, simulations of the model for cell 2018-06-25-ad are shown (see Table in “leaky integrate-and-fire models” section and Data S1 for parameters). (A_i_) Waveform (left) and corresponding power spectrum (right, same reference for decibel scale for all spectra shown in top two rows and a different reference for the spectra of the third row) of two superimposed cosine waves. No non-linearity is applied (green identity line in box). (A_ii_) Passing the signal through a dendritic low-pass filter, Eq. (A.4), here $\tau_{d}=1.88$ ms, attenuates the two frequencies. (A_iii_) A leaky integrate-and-fire neuron (LIF) does not encode the beating amplitude modulation in its spike train. (A_iv_) The height of the spike-train spectrum at the beat frequency grows weakly with beat amplitude α. (B) Applying a threshold (blue curve in threshold box) generates a peak in the power spectrum at the beat frequency (arrow), if the stimulus frequency $\omega_{2}$ is close to the carrier frequency $\omega_{1}$. This frequency component becomes more apparent after dendritic low-pass filtering and the LIF is able to generate spikes at the peaks of the beat. The spike times contain a spectral peak at the beat frequency (arrow) and the size of this peak strongly depends on beat amplitude. (C) For a stimulus frequency close to twice the carrier frequency, however, a threshold does not produce a spectral peak that would represent the beating envelope. After the dendritic low-pass filter the waveform still oscillates symmetrically around a fixed mean value and the LIF responds with tonic spiking that is not modulated by the signal’s envelope. (D) A cubed threshold (red curve in threshold box), however, generates a peak at the envelope frequency (arrows). Dendritic low-pass filtering results in an oscillating signal that shifts up and down according to the signal’s envelope. Consequently, the LIF is able to encode this envelope in its spiking activity and is also sensitive to the amplitude of the envelope.

Eventually, a spike generator, here modeled as a leaky integrate-and-fire neuron (LIF) with adaptation current, encodes the extracted envelope in a train of action potentials (Figure 7). LIF models with a threshold non-linearity without exponent (power of one), Equations 6, 7, 8, and 9,^38^ were individually fitted to baseline and step-response characteristics of n=9 recorded cells (See Table in “leaky integrate-and-fire models” section).

The non-linear spiking dynamics on its own is not sufficient to extract and respond to a beating AM resulting from two spectrally close frequencies. Although some models generate a peak at the beat frequency, their responses are almost independent of stimulus amplitude (Figure 7A).

A threshold non-linearity generates a sufficiently large spectral peak at the difference frequency. The spike generator is driven by this frequency and generates action potentials that encode the beat. Thresholding the signal results in modulations of the evoked firing rate responses that depend strongly on beat amplitude, faithfully reproducing the spiking properties of P-units (Figure 7B).

For stimulus frequencies close to twice the carrier frequency, however, action potentials do not encode the apparent envelope of the signal, because after thresholding and low-pass filtering, the input to the spike generator does not provide a frequency component at the envelope frequency as input (Figure 7C).

As worked out above, it requires a power of three applied to the thresholded signal to make the spike generator respond to the envelope at stimulus frequencies close to twice the carrier frequency (Figure 7D). Neither the hard threshold non-linearity the LIF applies on the membrane voltage nor the smooth voltage-threshold, of the exponential integrate-and-fire neuron (EIF, data not shown,^39^) can replace the power of three to extract the envelope resulting from a stimulus at around twice the carrier frequency. The non-linear dynamics of a spike generator cannot generate the aliasing structure of the P-unit responses. Rather, a sufficiently strong static non-linearity (Figure 7D_i_) has to be applied to the signal, such that the necessary low-frequency peak in the spectrum is generated. Subsequent low-pass filtering then isolates this peak (Figure 7D_ii_) and this is what the spike generator responds to.

### A power of three describes P-unit responses best

**Figure 8:**
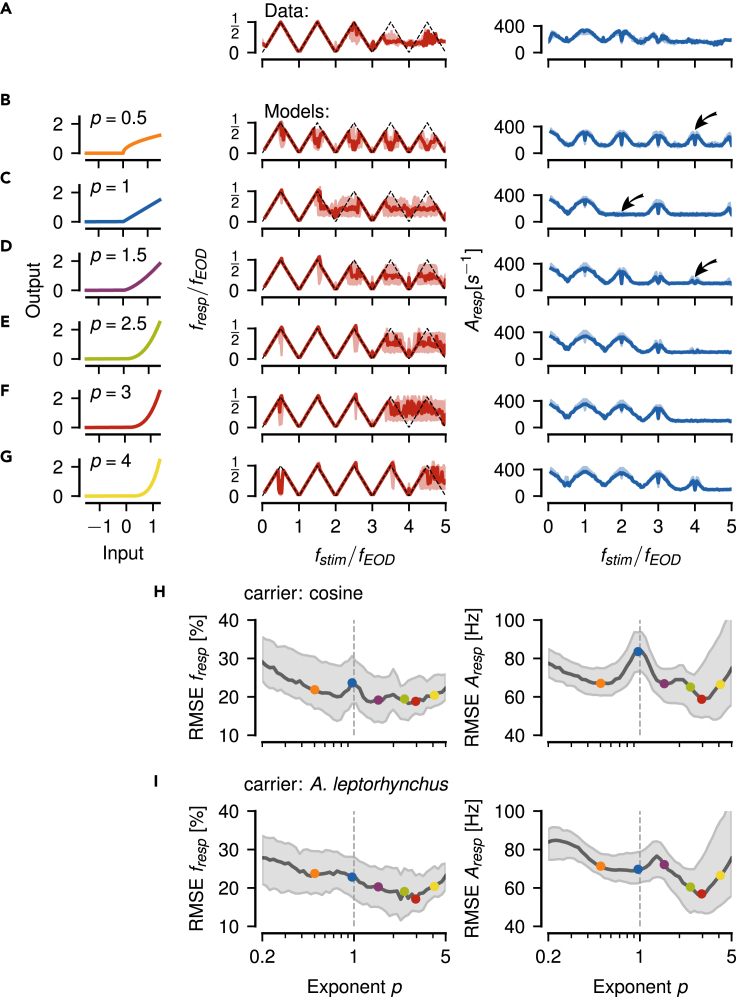
Dependence of LIF model performance on threshold exponent (A) Frequency (left) and amplitude tuning curves (right, population medians with interquartile ranges) of n=14 experimentally measured P-units of highest sensitivity (same as Figures 3B and 3C). (B–G) Tuning curves simulated from a population of n=9 LIF models (Figure 7) that have been fitted to individual P-units. Exponent p of the threshold non-linearity applied to the signal as indicated and illustrated in the left column. Arrows indicate missing or additional responses in the amplitude tuning curve. (H) Root mean squared error (RMSE, median with interquartile range) between the tuning curves of each experimentally measured P-unit shown in A and each LIF model in dependence on the threshold exponent used in the models. Examples from B–G are indicated by the correspondingly colored circles. Here, both the carrier and the stimulus are pure sine waves. (I) Same as in H but with an EOD waveform of *A. leptorhynchus* as carrier and a pure sine wave as stimulus, resembling the situation in the electrophysiological experiments (see Figure 9).

For a more systematic evaluation which exponent on the threshold operation describes the P-unit responses best, we simulated the LIF models using exponents at the threshold non-linearity ranging from p=0.2 to 5. The resulting frequency and amplitude tuning curves were compared to the experimentally measured ones (Figure 8).

As predicted, a pure threshold without exponent shows responses at the zeroth, first and third harmonic, but diverges from the measured activity (Figure 8A) around the second harmonic (Figure 8C). Exponents both higher (Figures 8D–8G) and lower than one (Figure 8B) fill in the response at the second multiple of $f_{EOD}$. Models with powers of 0.5, 1.5, and 4 additionally respond to the fourth multiple of $f_{EOD}$.

To quantify the model performance, we computed root mean squared errors (RMSE) between all pairings of the 14 experimentally measured cells with the 9 model cells of similar sensitivity. The RMSEs between frequency tuning curves were minimal at powers of about 0.8, 1.5, and 3 (Figure 8H, left). The RMSEs for the corresponding amplitude tuning curves showed similar minima but with the smallest RMSE at a power of three (Figure 8H, right). A power of three indeed describes both the frequency and amplitude tuning curves of P-unit responses best.

### Harmonics of the carrier are not sufficient to explain aliasing

**Figure 9:**
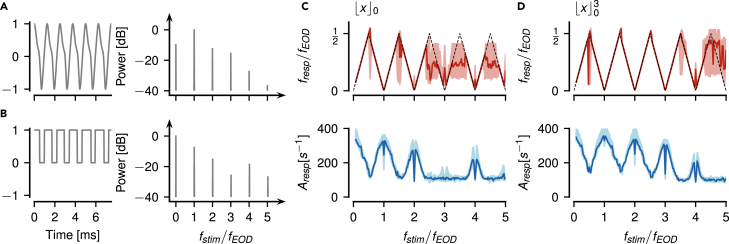
Influence of higher harmonics of the carrier on aliasing (A) An EOD waveform and the corresponding power spectrum of an *A. leptorhynchus* used as a carrier signal of frequency $\omega_{1}$ for simulating P-unit responses with the LIF models from Figure 8. (B) The corresponding pulse train (left), needed to approximate the threshold operation, Equation 2, has a duty cycle larger than 50%. Its spectrum (right) has a peak at the second multiple of $f_{EOD}$ in contrast to the spectrum of a pulse train with a 50% duty cycle (Figure 5B). (C) The enlarged duty cycle modifies the aliasing structure of the resulting frequency (top) and amplitude (bottom) tuning curves (medians with interquartile ranges of 9 simulated P-units), when using a threshold without exponent. For the example EOD waveform shown, the third harmonics and not the second as for a pure cosine wave is missing. (D) Taking in addition the thresholded signal to a power of three makes the tuning curves more independent of the duty cycle, an in particular fills in responses around three multiples of $f_{EOD}$.

So far, our reasoning was based on pure sine waves. In the electrophysiological recordings, however, the carrier was a real EOD waveform of *A. leptorhynchus*. Using these EOD waveforms instead of sine waves for the models made the minimum at an exponent of p=3 more distinct (Figure 8I). In a model with a pure threshold (p=1) the harmonics of the carrier do not contribute to shift the stimulus frequency to all multiples of $f_{EOD}$. A wider or narrower EOD waveform, however, modifies the aliasing structure introduced by the threshold operation in a way we do not observe in the data. Adding a power of three to the threshold makes the P-unit responses more robust against changes in the EOD waveform (Figure 9, see sections 6 and 7 in Data S1 for a more detailed explanation).

### Beats with the same envelope frequency evoke similar behavioral responses

**Figure 10:**
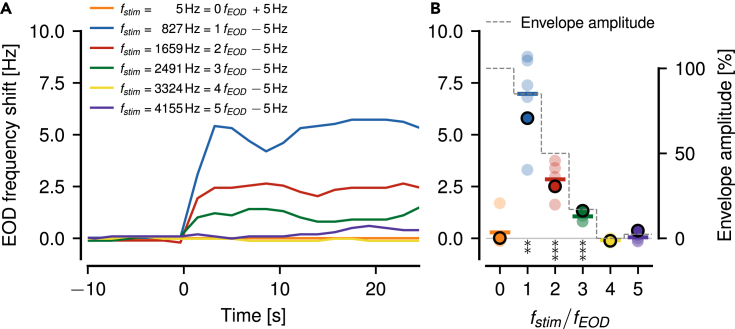
Jamming Avoidance Response (JAR) to stimuli close to multiples of $f_{EOD}$ (A) Shifts in EOD frequency of a single example fish induced by sinusoidal stimuli switched on at 0 s. The stimulus frequency was set 5 Hz above or below 0 to 5 times the EOD frequency of the fish as indicated. The fish responded to this stimulus by shifting its EOD frequency within about 10 s to a higher value. (B) Steady-state frequency shifts measured between 15 and 25 s after stimulus onset of five fish as a function of $f_{EOD}$ multiple. Frequency shifts of the fish shown in A are highlighted by circles with black outline. The frequency shifts averaged over the five fish (colored horizontal lines) approximately follow the corresponding envelope amplitudes predicted by a cubed threshold (dashed line, see section 4 in Data S1 for equations) for non-zero multiples of $f_{EOD}$. Frequency shifts in response to low absolute stimulus frequencies are mostly absent, despite having the largest envelope amplitude. Asterisks indicate significant deviations of the steady-state frequency shifts from zero (t-Test, ∗∗p<1 %, ∗∗∗p<0.1 %).

Our results imply that P-unit responses are potentially indistinguishable with respect to the absolute stimulus frequency, because P-unit responses to similarly mistuned multiples of $f_{EOD}$ differ only in modulation depth of their firing rate responses. We tested this hypothesis behaviorally by means of the jamming avoidance response (JAR,^25^). When a receiving fish is stimulated with a sinusoidal mimic that is close to but below the own EOD frequency, it will raise its EOD frequency by a few Hertz on a timescale of about 10 s (Figure 10A). We repeated the experiment with stimulus frequencies 5 Hz below one to five times $f_{EOD}$. Indeed, all the fish tested (n=5) responded with a significant increase of their EOD frequency to stimulus frequencies close to one, two, and three multiples of their EOD frequency (Figure 10B). None of the fish responded to four times $f_{EOD}$, but some fish slightly elevated their EOD frequency in response to five times $f_{EOD}$ by less than 1 Hz. The size of the frequency shift in response to non-zero multiples of $f_{EOD}$ approximately follow the amplitudes of the corresponding envelopes predicted by a cubed threshold (Figure 10C). To the stimulus at the zeroth multiple of $f_{EOD}$ only a single fish responded with a noticeable frequency shift.

## Discussion

We observed that P-units respond over a wide range of stimulus frequencies up to about 3000 Hz (Figure 1). This does not mean, however, that P-units unambiguously encode, for example, a 3000 Hz stimulus frequency. Rather, P-units are driven by the summation of the self-generated EOD and an exogenous stimulus, resulting in a beating signal envelope. At integer multiple of the carrier frequency these envelopes are slow and between integer multiples they are fast, a pattern reminiscent of aliasing known from the sampling theorem. In accordance with these reoccurring envelopes, the P-unit tuning curves are also periodic in multiples of the EOD frequency, whereas the amplitude of the responses declined with higher stimulus frequencies.

### P-unit tuning to high difference frequencies

P-unit tuning curves for beat stimuli have so far been only measured for difference frequencies up to about 300 Hz.^17^^,^^18^^,^^22^^,^^23^ The amplitude of the firing-rate modulation induced by beating envelopes resembles a band-pass tuning: At frequencies close to zero the P-unit responses are reduced^18^ which reflects the high-pass filter induced by fast spike-frequency adaptation.^19^ Toward higher beat frequencies the response was found to steadily decline toward zero as expected for a spiking neuron^39^ and as set by the neuron’s baseline firing rate.^40^

The experimental findings reported here clearly demonstrate that P-units do show responses to difference frequencies beyond 300 Hz. Instead of the expected steady decline, the tuning repeats at multiples of the EOD frequency. This has profound consequences for the encoding of electrocommunication signals, so-called chirps, that transiently change the difference frequency.^19^^,^^22^ The P-unit response to chirps could be explained by transient firing rate modulations mediated by the P-units’ amplitude tuning curve.^23^ In a field study the behavioral relevance of chirps at difference frequencies beyond 400 Hz and thus beyond $f_{EOD}/2$ of the female has been observed.^24^ A monotonously declining tuning curve would not suffice to explain how chirps could be encoded at such high difference frequencies. The repetitive tuning we describe here retains the capability to change the modulation depth of the firing rate in response to chirps.

In this context, the low-pass filtering happening at the synapse between afferents and their targets, the pyramidal cells in the electrosensory lateral line lobe, plays an important role (Figure 2). If the kernel is too narrow, then the tuning curve of the P-units is almost flat and firing rate modulations caused by chirps would be quite small. If the kernel is too wide, the tuning curve is only modulated within a narrow range of stimulus frequencies around multiples of the carrier frequency. Only for kernels resembling the experimentally measured postsynaptic potentials (σ=0.5 ms,^31^) is the tuning curve fully modulated without being flat between the multiples (Figure 3). We therefore hypothesize electric fish with lower EOD frequencies, like for example *Eigenmannia* spec., to have correspondingly wider postsynaptic potentials.

Surprisingly, we observed P-unit responses down to absolute stimulus frequencies of about 35 Hz, a range commonly assumed to be primarily driving the ampullary electrosensory system.^32^^,^^41^^,^^42^ Toward even lower stimulus frequencies responses vanish, although there seems to be a large variability between cells (not shown). Future recordings with a finer resolution of the stimulus frequencies are needed for in depth exploration of this specific frequency range.

### Envelope extraction at high difference frequencies

The repetitive tuning curve of P-units is in a way trivial in that it simply follows the envelopes visible in the superimposed cosine signals. But how are these envelopes extracted from the original signals, that do not contain the envelope frequencies in their power spectra?

Usually, the envelope frequency is considered equal to the difference frequency.^20^^,^^23^ We have shown that extracting the envelope by the Hilbert transform or squaring of the signal^33^^,^^34^^,^^43^ fails for higher difference frequencies and are not sufficient to explain the experimental observations (Figure A1A and A1B in Data S1). Both lead to spectral peaks that reflect the true envelope for frequency differences below half the carrier frequency but fail beyond. Furthermore, for stimulus frequencies close to even multiples of the carrier frequency, the resulting signals cannot be described as AMs, because their lower and upper envelope are out of phase (Figure 4A).

For analyzing EEG/EMG^44^ or acoustic signals^45^ simple thresholding is often applied to compute envelopes. Such a threshold, in deep learning also known as a Rectifying Linear Unit (ReLU), extracts envelope frequencies only at odd harmonics of the carrier (Figure 5). Our results show that a threshold operation followed by exponentiation is required to explain the experimental findings (Figures 6 and 8). Neither the non-linearity of action-potential generation (Figure 7), nor higher harmonics of the carrier and the signal are sufficient to substitute the exponentiated threshold (Figure 9).

Of interest, it has become common to apply smooth threshold functions such as ELU^46^ or Softplus^47^ in deep learning approaches using artificial neural networks. All of these are potential alternatives for the threshold raised to a power of three we are suggesting here, and can be approximated by a ReLU raised to a power of three in the vicinity of their threshold. The same holds true for sigmoidal activation functions which are discussed for the transformation of a hair-cells membrane voltage by their ribbon synapse.^37^ Only for larger inputs their saturation will lead to noticeable deviations.

### Extraction of secondary envelopes

Secondary envelopes, the modulation of the amplitude of beats, arise from relative movements between two fish^10^ and interactions between more than two fish.^34^^,^^48^ They provide a context onto which electrocommunication signals are encoded in the thalamus.^49^ Secondary envelopes, however, are mainly extracted downstream of P-units in the ELL^48^ by means of threshold non-linear response curves of the involved neuron.^33^ The input from which the envelopes are extracted are not superpositions of sine waves anymore, but rather temporally modulated population firing rates. Therefore, the problem of encoding high stimulus frequencies does not exist in the context of encoding secondary envelopes. However, our results demonstrate that secondary envelopes are also to be expected on slow beating envelopes arising close to multiples of the carrier frequency and that these will also be encoded in the electrosensory system.

### Sinusoidal amplitude modulations (SAMs) versus beats

SAMs of various frequencies have been used to characterize signal processing in the electrosensory^17^ and the mammalian auditory system.^20^ SAM stimuli multiply a carrier — the EOD of an electric fish or a tone — with a periodic amplitude modulation, like in Equation S6, and differ from superimposed cosines, Equation 1, by having three spectral peaks instead of two. The additional side-peak of a SAM stimulus already fills in responses at the second multiple of the carrier frequency when used in conjunction with a threshold without exponent (Figure A2C and section 5 in Data S1). This effect would have obscured the necessary cubed threshold, if we had used SAMs instead of realistic superimposed cosines.

### Relation to the sampling theorem

In the limit to an infinitely high exponent, the thresholded and exponentiated carrier approaches Dirac delta functions positioned at multiples of the carrier’s period. This pulse train can be thought to sample the stimulus waveform with the carrier frequency exactly like in the setting of the sampling theorem. However, the stimulus waveform also gets transformed by the threshold and the exponentiation. The higher the exponent the larger the distortion of the extracted envelope. Thus, the exponent should not be too large to maintain an accurate representation of the amplitude modulation. In this sense, a sharp threshold without exponent would be ideal.

### Physiological mechanisms for beat extraction

Our theoretical considerations suggest an exponent of at least three. What are possible physiological substrates of such a non-linear operation?

First, we need to note that a perfect threshold is a mathematical abstraction. Any physiological mechanism implementing a threshold operation has a smooth transition. A cubic power applied to the threshold approximates such a smooth threshold (Figure 7D) and in that sense can be considered biologically more plausible than a perfect threshold.

The most likely site for the smooth threshold operation are the ribbon synapses of the electroreceptor cells onto their afferents^14^^,^^15^ as has been suggested previously.^50^ The sigmoidal shape of the activation function of voltage-gated calcium channels that trigger synaptic transmitter release, for example, implement a threshold operation. Unfortunately, the synapse connecting primary electroreceptors and the afferents are difficult to access and there are no recordings of their transfer function.

One afferent receives input from a set of primary electroreceptors. Before the postsynaptic potentials reach the spike-generation site they are likely low-pass filtered by passive dendritic conduction.^38^ With the right time-constant this isolates the low-frequency amplitude modulation but does not entirely remove the EOD (Figure 7, second row). This is supported by the phase locking of P-unit spikes to the EOD. The vector strength quantifying this phase-locking is well below one.^32^ It would be expected to be much closer to one without low-pass filtering before the spike-generator. With a stronger low-pass filter the P-units would lose their locking to the EOD.

P-units are also tuned to a fish’s own EOD frequency as has been shown by silencing the EOD and probing the P-unit response to varying artificial EOD frequencies.^4^ P-units are most sensitive to the fish’s EOD frequency and close-by stimulus frequencies (Figure A3A in Data S1). The corresponding band-pass filter is probably caused by electric resonance in the electroreceptor cells.^51^ Adding a damped oscillator, Equation S27, to our P-unit models, (Equation 6), (Equation 7), (Equation 8), (Equation 9), reproduces P-unit tuning to EOD frequency (Figures A3A and A3B in Data S1) but does not impair responses to beats at high difference frequencies (Figure A3C and A3D in Data S1).

### Ambiguity in beat perception

The decline in response amplitude with multiples of the carrier (Figure 3) results from the declining envelope amplitude (Figure 6E). Because the envelope amplitude also depends on the distance between two fish — the larger the distance the smaller the envelope amplitude^10^ — it cannot be used to disambiguate different multiples of $f_{EOD}$. Therefore, P-unit responses to similarly mistuned multiples of $f_{EOD}$ are ambiguous and the fish should not be able to resolve absolute stimulus frequency based on the firing rate modulation of P-units.

The jamming avoidance response (JAR) is evoked by stimulus frequencies close to the receiver’s own EOD frequency and also twice its EOD frequency.^25^ Here we report JARs even at the third multiple of the EOD frequency. The EOD frequency shift of the JAR approximately follows the corresponding envelope amplitude as extracted by a cubed threshold up to five times the EOD frequency (Figure 10). Furthermore, the behavioral threshold for detecting a stimulus frequency^52^ at least qualitatively follows the repetitive tuning curve of the P-units reported here. This suggests that wave-type electric fish indeed cannot disambiguate stimuli at different multiples of their EOD frequency.

In contrast, most fish did not respond to a stimulus frequency close to the zeroth multiple of $f_{EOD}$, although there the envelope amplitude is largest. However, also the P-unit response vanished at stimulus frequencies below about 35 Hz (Figure 1A, 1J and 3C). Such low-frequency stimuli also evoke responses in ampullary cells of the passive electrosensory system^32^^,^^41^^,^^42^ that would allow the fish to disambiguate the (weak) P-unit responses and to inhibit the JAR response at such low stimulus frequencies.

### Perception of other wave-type species

The wide range of difference frequencies covered by the P-units (Figure 3) extends far beyond the range of EOD frequencies observed in conspecific wave-type electric fish (usually about one octave). Consequently, the fish should be able to detect the presence of sympatric species that signal in higher or lower frequency ranges.^9^^,^^53^^,^^54^^,^^55^^,^^56^ Whether and how the different species interact or communicate remains an open question that could be resolved by analyzing electrode-array data recorded in the field.^9^

### Beat perception in the auditory system

Early psycho-physical experiments with interacting pure tones demonstrated that beats are not only perceived at low difference frequencies but also for mistuned octaves, when the second tone is close to octaves of the first tone.^5^^,^^26^ These experimental findings were formalized in the 19th century by Ohm^27^ and Helmholtz.^28^ Beats at higher octaves are better perceived the lower the frequency of the carrier and the louder the signal.^6^ Furthermore, masking experiments ruled out aural harmonics and interactions with combination tones as possible mechanisms underlying beat perception.^6^ Our observations of clearly modulated firing rate responses at mistuned octaves suggest threshold non-linearities within auditory fibers as a potential mechanism.

### Non-linear physiological mechanisms in the mammalian auditory periphery

Distortion-product otoacoustic emissions (DPOEs) are a hallmark of non-linear phenomena in the ear that have been attributed to the mechanical properties of the cochlea and in particular the active amplification of outer hair cells.^57^ The most prominent DPOEs are the quadratic distortion, $\omega_{2}-\omega_{1}$, which is the difference frequency, and the cubic distortion, $2\omega_{1}-\omega_{2}$.^58^ They could explain beat-like responses to stimuli close to one and two multiples of a tone, but not to higher multiples.

The focus of auditory neuroscience has been on the encoding of SAMs,^20^ often in neurons with quite high characteristic frequencies, such that only the initial declining part of the temporal modulation transfer functions have been recorded.^59^ A neuronal correlate of beat perception at high difference frequencies is still not known. Our findings in the electrosensory system predict that a smooth threshold operation within a single auditory nerve fiber could generate distortion products needed to extract the aliasing structure of signal envelopes.

Both the sigmoidal mechanosensory transducer function^60^ as well as the transfer function of the hair-cell ribbon synapse^61^ are good candidates for such a non-linear transformation. In particular, cooperativity of calcium channels in the presynapse has been discussed for hair cells in the auditory system to result in powers of three or higher.^35^^,^^36^^,^^37^

The two superimposed tones need to enter the hair cell with sufficient amplitudes for these mechanisms to take effect. The lower the characteristic frequency of an auditory fiber and the louder the two tones, the wider its effective tuning,^62^^,^^63^ potentially allowing for superimposed tones that differ by multiple octaves to interact within a single auditory fiber. This is in line with the frequency and intensity dependence of beat perception discussed above.^6^

For testing our hypothesis, single auditory fibers should be stimulated with two tones centered symmetrically within their tuning curves. The resulting frequency and amplitude tuning curves allow then to deduce the effective power on the threshold operations implemented in the auditory fiber (Figure 8). Knock-outs of outer hair cell function or manipulations of the hair cell synapses would then allow to assess the respective contributions.

### Conclusions

A threshold operation with its sharp edge is a mathematical abstraction. Any physiological mechanism implementing this non-linearity, like for example the activation curve of voltage-gated calcium currents or the transfer function of a synapse, has a rather smooth transition. The cubed threshold we derive from our recordings is a mathematically simple way for modeling such a physiologically realistic smooth threshold. In this sense, the ability of the P-units to extract beats at multiples of the carrier frequency is an epiphenomenon of their physiology. For the same reason, mammalian auditory fibers are bound to respond to mistuned octaves and thus should contribute to the percept of beats at higher difference frequencies.

### Limitations of the study

The behavioral observations regarding the jamming avoidance response (JAR) are based on a single difference frequency. Future studies should check for a whole range of difference frequencies relative to the integer multiples of the EOD frequency to analyze the full JAR profile. This is especially important for analyzing the behavior at low absolute stimulus frequencies.

## STAR★Methods

### Key resources table

**Table:** 

REAGENT or RESOURCE	SOURCE	IDENTIFIER
**Deposited data**		

P-unit recordings	https://g-node.org	https://doi.org/10.12751/g-node.gn1ll1

**Experimental models: Organisms/strains**		

*Apteronotus leptorhynchus*	Aquarium Glaser GmbH, Rodgau, Germany	N/A

**Software and algorithms**		

nixio	Stoewer et al.^64^	RRID:SCR_016196
thunderfish	https://github.com/bendalab/thunderfish	N/A

### Resource availability

#### Lead contact

Further information and requests for resources and code should be directed to and will be fulfilled by the lead contact, Jan Benda (jan.benda@uni-tuebingen.de).

#### Materials availability

This study did not generate new unique materials.

### Experimental model and subject details

Experiments were performed on the male and female weakly electric fish of the species *Apteronotus leptorhynchus* obtained from a commercial tropical fish supplier (Aquarium Glaser GmbH, Rodgau, Germany). The fish were kept in tanks with a water temperature of 25° C and a conductivity of around 270μS/cm under a 12 h:12 h light-dark cycle. Body sizes of the fish were between 15 and 17.5 cm and 11.1 and 13.2 g. $f_{EOD}$ varied between 558 and 860 Hz. All experimental protocols complied with national and European law and were approved by the Ethics Committee of the Regierungspräsidium Tübingen (permit no: ZP1-16).

### Method details

#### Surgery

Prior to surgery, anesthesia was provided via bath application of a solution of MS222 (120 mg/l, PharmaQ, Fordingbridge, UK) buffered with Sodium Bicarbonate (120 mg/l). For the surgery the fish was fixed on a stage via a metallic rod glued to the skull. The posterior anterior lateral line nerve (pALLN) above the gills, before its descent towards the anterior lateral line ganglion (ALLNG) was disclosed for subsequent P-unit recordings. During the surgery water supply was ensured by a mouthpiece, sustaining anesthesia with a solution of MS222 (100 mg/l) buffered with Sodium Bicarbonate (100 mg/l).C.

#### Experimental setup

Fish were immobilized by an initial intramuscular injection of Tubocurarine (Sigma-Aldrich, Steinheim, Germany; 25–50 μL of 5 mg/mL solution). For the recordings fish were fixated on a stage in a tank, with a major part of the body immersed in water. Analgesia was refreshed in intervals of two hours by cutaneous Lidocaine application (2 %; bela-pharm, Vechta, Germany) around the operation wound and the head mounting rod. Electrodes (borosilicate; 1.5 mm outer diameter; GB150F-8P; Science Products, Hofheim, Germany) were pulled to a resistance of 50–100 MΩ (model P-97; Sutter Instrument, Novato, CA) and filled with 1 M KCl solution. Electrodes were fixed in a microdrive (Luigs-Neumann, Ratingen, Germany) and lowered into the nerve. Recordings of electroreceptor afferents were amplified (SEC-05, npi-electronics, Tamm, Germany, operated in bridge mode). All signals, P-unit recording, local and global EOD (see below) and the generated stimulus, were digitized with a sampling rate of 40 kHz (PCI-6229, National Instruments, Austin, TX). RELACS (www.relacs.net) running on a Linux computer was used for online spike and EOD detection, stimulus generation, and calibration.

#### P-unit identification

P-units were identified based on their firing properties with a baseline firing rate between 64–530 Hz,^32^^,^^65^^,^^66^ phase locking to the EOD, indicated by multimodal interspike-interval (ISI) histograms, and by responses to amplitude modulations of the EOD.

#### Electric field recordings

Global EOD for monitoring $f_{EOD}$ was measured with two vertical carbon rods (11cm long, 8 mm diameter) in a head-tail configuration. The signal was amplified 200–500 times and band-pass filtered (3 to 1 500 Hz passband, DPA2-FX; npi electronics, Tamm, Germany). A local EOD including the stimulus was measured between two, 1 cm-spaced silver wires located next to the left gill orthogonal to its longitudinal body axis (amplification 200–500 times, band-pass filtered with 3 to 1 500 Hz pass-band, DPA2-FX; npi-electronics, Tamm, Germany). Unfortunately, these filter settings were too narrow for the high stimulus frequencies we used. For Figures 1A–1F we recreated the local stimulus waveforms by adding the recorded stimulus output to the global EOD to avoid unwanted phase shifts.

#### Stimulation

Sine wave stimuli (10–3300 Hz) imitating another fish were isolated (ISO-02V, npi-electronics, Tamm, Germany) and delivered via two horizontal carbon rods located 15cm laterally to the fish. Depending on $f_{EOD}$ of the fish, the stimuli resulted in difference frequencies between −750 and 2495 Hz. Each stimulus was repeated twice either for 0.5 s (20% of the trials) or 1 s (80% of the trials). Stimulus amplitude was fixed at 10% or 20% of the fish’s local EOD amplitude (contrast) measured prior to each stimulation. From cells stimulated with both amplitudes we only considered the amplitude with the larger number of stimulus frequencies tested for further analysis, such that each cell contributed only once to the population analysis.

#### Jamming-avoidance response

For measuring the jamming avoidance response we placed a fish in a 40×50 cm^2^ tank filled with water from their home tank (∼250 μS/cm conductivity) to a height of 20 cm, where they voluntarily stayed in a plastic tube. With the same techniques and equipment as for the electrophysiology we measured the fish’s EOD frequency via two carbon electrodes placed in front and behind the tube where the fish was hiding. The fish were stimulated with another pair of carbon electrodes placed orthogonal to the measurement electrodes to the side of the fish about 10 cm apart. Within a time window of 10 seconds the EOD frequency of the fish was estimated from a power spectrum right before each stimulus presentation. Sinewave stimuli were calibrated to an amplitude of 2 mV/cm measured at the position of the fish. Stimulus frequencies were fixed for the 30 sec long duration (no frequency clamping) and set to k times $f_{EOD}$ minus 5 Hz for 1≤k≤5 and +5 Hz for k=0, with $f_{EOD}$ measured right before stimulus onset. Each stimulus frequency was presented once to each fish.

From the recorded fish’s EOD we computed a spectrogram (FFT segment length of n=131072 samples with an overlap of 50 %). The time course of the EOD frequency was estimated from the peak frequency of the second harmonics in the spectrogram. To compute the frequency shift we subtracted the baseline EOD frequency estimated as the averaged EOD frequency within 10 s right before stimulus onset. Steady-state frequency shift was estimated as the average EOD frequency within 15 to 25 s after stimulus onset relative to baseline EOD frequency.

### Quantification and statistical analysis

#### Sample size

40 P-units were recorded from seven weakly electric fish of the species *Apteronotus leptorhynchus*.

#### Data analysis

Data analysis was performed with Python 3 using the packages matplotlib, numpy, scipy, sklearn, pandas, nixio,^64^ and thunderfish (https://github.com/bendalab/thunderfish).

In binary spike trains with a time step of 0.025 ms each spike was indicated by a value of 40 kHz and all other time bins were set to zero. Time-resolved firing rates were computed by convolving the spike trains with a Gaussian kernel. The standard deviation of the kernels was set to σ=0.5 ms or σ=2 ms. In the frequency domain, these kernels are also Gaussians centered at zero frequency and with a standard deviation of $\sigma_{f}={\left(2\pi \sigma \right)}^{-1}=318$ Hz or $\sigma_{f}=80$ Hz, respectively. Convolution with kernels corresponds to constructing peri-stimulus time histograms but avoids edge effects introduced by the histogram bins.

Power spectra of binary spike trains or time-resolved firing rates in response were computed from fast Fourier transforms on $n_{\text{fft}}=4096$ long data segments that overlapped by 50 %. The initial and last 5 ms of each spike train were excluded from the analysis.

Frequency tuning curves, the position $f_{resp}$ of the largest peak in the power spectrum of the time-resolved firing rate as a function of stimulus frequency $f_{stim}$, tell us on which frequency the firing rate was modulated by the stimulus. The corresponding amplitude of this peak, $A_{resp}$, estimated as the square root of the integral of the power spectrum over the five frequencies closest to the peak frequency, quantifies the modulation depth of this firing rate modulation. Amplitudes of spectral peaks are closely related to the vector strength that is commonly used to quantify temporal modulation transfer functions of auditory neurons in response to SAMs.^38^ Baseline firing rate was calculated as the number of spikes divided by the duration of the baseline recording (on average 18 s).

During each stimulus presentation we estimated $f_{EOD}$ from the recorded global EOD as the frequency of the largest peak in power spectra computed with $2^{16}$ samples per FFT window. In the same way we also confirmed the stimulus frequency. Difference frequencies and stimulus frequencies relative to $f_{EOD}$ were then reported based on these measurements.

Cells with less than 50 different stimulus frequencies, cells with no stimulus frequencies higher than $2.6f_{EOD}$ and cells with no difference frequencies between 0 and 100  Hz were excluded from the analysis. Stimulus frequencies resulting in envelopes of periods larger than the analysis window were also excluded from the analysis.

To estimate how far cells follow the aliasing structure to higher stimulus frequencies ($f_{\max}$ in Figure 3 A), we computed for each stimulus frequency $f_{stim}$ the quadratic deviation $\sigma ={\left(f_{resp}/f_{EOD}-f_{\exp}/f_{EOD}\right)}^{2}$ of the measured response frequency $f_{resp}$ from the expected alias frequency of the stimulus $f_{\exp}=\left|f_{stim}-f_{EOD}\left\lfloor f_{stim}/f_{EOD}\right\rfloor \right|c$, where ⌊x⌋ rounds x to the closest integer. We then looped over all stimulus frequencies $f_{stim,i}$ and added up the corresponding bins $\left(f_{stim,i+1}-f_{stim,i-1}\right)/2$ of the stimulus frequencies, but only if the response frequency matched the expected one (σ<0.0005). This sum, $f_{\max}$, quantifies the range of stimulus frequencies for which the response of the cell follows the expected alias frequencies.

#### Leaky integrate-and-fire models

We constructed leaky integrate-and-fire (LIF) models to reproduce the specific firing properties of P-units^38^^,^^50^:

(Equation 6) $$\tau_{m}\frac{dV_{m}}{dt}=-V_{m}+f\left(V_{m}\right)+\mu +\beta V_{d}-A+\sqrt{2D}\xi$$

where $\tau_{m}$ is the membrane time constant, μ a bias current, and D is the strength of Gaussian white noise ξ. Whenever the unitless membrane voltage $V_{m}$ crosses the threshold of θ=1, a spike is generated and the voltage is reset to $V_{m}=0$.

The static non-linearity $f\left(V_{m}\right)$ equals zero for the LIF. In case of an exponential integrate-and-fire model (EIF), this function was set to,^39^

(Equation 7) $$f\left(V_{m}\right)=\Delta_{V}e^{\frac{V_{m}-1}{\Delta_{V}}}$$

where we varied $\Delta_{V}$ from 0.001 to 0.1.

The prominent spike-frequency adaptation of P-units^19^ is modeled by an adaptation current A with dynamics

(Equation 8) $$\tau_{A}\frac{dA}{dt}=-A$$

and adaptation time-constant $\tau_{A}$. Whenever a spike is generated, the adaptation current is incremented by $\Delta_{A}$.^67^

The input to the LIF is the membrane voltage $V_{d}$ of a dendritic compartment scaled by β, that low-pass filters the rectified, Equation 2, electrosensory stimulus x(t) with a time constant of $\tau_{d}$:

(Equation 9) $$\tau_{d}\frac{dV_{d}}{dt}=-V_{d}+{\left\lfloor x\right\rfloor }_{0}^{p}$$

This dendritic low-pass filtering was needed for reproducing the loose coupling of P-unit spikes to the EOD, while maintaining high sensitivity to small amplitude modulations. The rectified stimulus was optionally taken to a power of p.

The stimulus is the EOD of the receiving fish normalized to an amplitude of one plus the EOD of a second fish. If not stated otherwise, a superposition of cosine waves, Equation 1, was used to mimic the EODs. Realistic EODs (Figure 8I) were generated by summing up the first 10 harmonics whose relative amplitudes and phases have been extracted from head-tail recordings obtained during measurements of P-unit baseline activity using our thunderfish software, https://github.com/bendalab/thunderfish.

The 8 free parameters of the P-unit model, $\tau_{m}$, μ, β, D, $\tau_{A}$, $\Delta_{A}$, $\tau_{d}$, and $t_{ref}$, were fitted to both baseline activity (baseline firing rate, CV of inter-spike intervals (ISI), serial correlation of ISIs at lag one, and vector strength of spike coupling to EOD) and responses to step in- and decreases in EOD amplitude (onset- and steady-state responses, effective adaptation time constant) of 9 specific P-units (See Table below1; Data S1) for a fixed power of p=1. When modifying the model (e.g. varying the threshold non-linearity or the powers p), we adapted the bias current μ to restore the original baseline firing rate.

**Table:** Model parameters, fitted to nine specific P-units

*cell*	β	τ_*m*_ /ms	μ	*D* / *ms*	τ _*A*_ /ms	Δ _*A*_	τ_*d*_ /ms	*t*_*ref*_ /ms
2012-04-20-ak	373.7	1.74	21.88	0.13	294.95	142.7	4.29	0.93
2011-10-25-ad	301.4	2.90	−34.38	0.44	116.08	60.7	4.08	0.74
2012-12-20-ab	46.6	1.35	−4.79	0.03	48.01	9.3	1.21	1.14
2012-12-20-ad	124.2	1.06	−16.21	0.06	93.17	23.0	4.54	1.09
2012-12-20-ae	190.1	1.65	−31.84	0.07	61.15	28.8	5.37	0.92
2012-12-21-ai	291.2	2.10	−54.69	0.55	127.44	36.8	3.15	1.20
2014-06-06-ac	382.9	4.98	−70.70	4.00	111.95	55.1	2.40	0.62
2018-05-08-af	266.9	1.19	−35.16	0.17	61.96	50.7	8.87	1.10
2018-06-25-ad	286.1	2.39	−29.69	2.44	96.35	62.5	1.88	1.09
median	286.1	1.74	−31.84	0.17	96.35	50.7	4.08	1.09

## Data Availability

• The P-units recordings generated in this study have been deposited at www.g-node.org and are publicly available as of the date of publication. DOIs are listed in the key resources table. • This paper does not report original code. • Any additional information required to reanalyze the data reported in this paper is available from the lead contact upon request.

## Supplemental information

Data S1. Mathematical derivations
