# Supplementary material for: Beat encoding at mistuned octaves within single electrosensory neurons
Source: iScience. 2023 May 13;26(7):106840. doi: 10.1016/j.isci.2023.106840 (PMC10331418; doi:10.1016/j.isci.2023.106840)
Supplement: Data S1. Mathematical derivations [file mmc2.pdf]

Mathematical derivations

1. Analytic signal

The analytic signal corresponding to the original signal is constructed by means of the Hilbert transform. With this method any signal can be expressed as a product

$$x(t) = A(t)\cos(\varphi(t)) \quad \text{(S1)}$$

where the amplitude modulation $A(t)$ is the absolute value of the analytic signal and $\varphi(t)$ is the phase of the analytic signal. The amplitude of the carrier $\cos(\varphi(t))$ is modulated by $A(t)$. Whereas the Hilbert transform itself is linear, taking the absolute value is a non-linear operation.

For the superimposed cosines, (1), we get for the amplitude modulation

$$A(t) = |x(t)| = \sqrt{1+\alpha^2 + 2\alpha\cos((\omega_2-\omega_1)t)} \quad \text{(S2)}$$

and for the phase

$$\varphi(t) = \frac{\omega_1+\omega_2}{2}t + \arctan\left(\frac{1-\alpha}{1+\alpha}\cdot\tan\left(\frac{\omega_1-\omega_2}{2}t\right)\right) \quad \text{(S3)}$$

[34]. This is an exact identity. The Hilbert transform is just a mathematical trick to transform any signal into such a product of an amplitude modulation and a cosine carrier.

For $\alpha = 1$ (both cosine waves have the same amplitude) this reduces to the well known identity

$$x(t) = 2\cos\left(\frac{\omega_2-\omega_1}{2}t\right)\cos\left(\frac{\omega_1+\omega_2}{2}t\right) \quad \text{(S4)}$$

A carrier signal of frequency $(\omega_1+\omega_2)/2$ is multiplied with an amplitude modulation with frequency $(\omega_1-\omega_2)/2$. The latter frequency is half the frequency of the beating amplitude modulation.

For small amplitudes $\alpha \to 0$ the expansion of the amplitude modulation to first order results in

$$A(t) \approx 1+\alpha\cos(\Delta\omega t) \quad \text{(S5)}$$

This amplitude modulation has a constant zero-frequency component in the Fourier spectrum, and one at the difference frequency $\Delta\omega = \omega_2 - \omega_1$. For larger amplitudes more and more harmonics of this peak appear.

This is exactly what we expect for low difference frequencies, i.e. for stimulus frequencies ω_2 close to ω_1. However, for higher difference frequencies, the amplitude of the analytic signal Eq. (S5) suggests that the beat frequency keeps increasing with increasing difference frequency, no matter how large the

difference frequency (Fig. A1 A). It does not explain the aliasing structure we observe in the signals and the P-unit responses. This does not imply that the analytic signal is wrong. Rather the amplitude term (S5) simply does not capture the obvious aliasing structure of the beats. It is hidden in the phase term Eq. (S3).

On a first glance, the phase of the carrier simplifies to $\varphi(t) = \omega_1 t$ for small amplitudes. However, this is valid only for $\alpha = 0$, because only then $\frac{1-\alpha}{1+\alpha} = 1$ in Eq. (S3). The resulting small-amplitude approximation

$$x(t) \approx (1 + \alpha \cos(\Delta\omega t))\cos(\omega_1 t) \quad \text{(S6)}$$

is in fact not a good approximation. In the Fourier spectrum it has two side-peaks at $\omega_1 \pm \Delta\omega$ instead of only one at $\omega_1 + \Delta\omega = \omega_2$ flanking the carrier at ω_1. Eq. (S6) no longer is a beat resulting from the superposition of two cosine waves, but a sinusoidal amplitude modulation (SAM). The approximation fails, because the $\frac{1-\alpha}{1+\alpha}$-term in Eq. (S3) quickly deviates from one with slope -2 as amplitude increases.

2. Squaring

An alternative method to retrieve amplitude modulations is to square the signal and then low-pass filter it. Squaring the beat (1), using the binomial theorem and the trigonometric power reduction formula results in

$$\begin{aligned} x^2(t) = \; & \frac{1}{2}(1+\alpha^2) + \frac{1}{2}\cos(2\omega_1 t) + \frac{1}{2}\alpha^2 \cos(2\omega_2 t) \\ & + \alpha \cos((\omega_2 - \omega_1)t) + \alpha \cos((\omega_1 + \omega_2)t) \end{aligned} \quad \text{(S7)}$$

While the original signal (1) has two peaks in the power spectrum at ω_1 and ω_2 and no peak at the beat frequency, the power spectrum of the squared signal (S7) has five peaks, one for each term (Fig. A1 B). Shifting and generating new peaks in the spectrum is a hallmark of non-linear operations. The squaring operation doubles the two original frequencies and creates a new high-frequency peak at the sum of the two frequencies. In addition, a new peak occurs at zero, representing the non-zero mean of the squared signal. Another peak appears at the difference frequency $\omega_2 - \omega_1$. This is the amplitude modulation. By subsequent low-pass filtering this peak can be isolated and that way the amplitude modulation can be retrieved. However, as for the analytic signal, none of the five terms explain the aliasing structure of the beat.

3. Thresholding

The Fourier spectrum of the pulse train, Eq. (4), turns out to have peaks at odd multiples of ω_1 with amplitudes

$$c_k = \frac{\omega_1}{2\pi} \int_{-\frac{\pi}{2\omega_1}}^{+\frac{\pi}{2\omega_1}} e^{-i\omega_1 kt}\, dt = \frac{1}{\pi k} \sin\left(\frac{\pi}{2} k\right) \quad \text{(S8)}$$
$$= \frac{1}{\pi k}(-1)^{\frac{k-1}{2}}, \; k \text{ odd}$$

and an additional peak at zero frequency with amplitude

$$c_0 = \frac{\omega_1}{2\pi}\left[t\right]_{-\frac{\pi}{2\omega_1}}^{+\frac{\pi}{2\omega_1}} = \frac{1}{2} \quad \text{(S9)}$$

Thresholding a cosine with the same frequency ω_1 can be approximated by multiplying the cosine with the pulse train Eq. (4):

$$\lfloor \cos(\omega_1 t) \rfloor_0 = \cos(\omega_1 t) \cdot p(\omega_1 t) \quad \text{(S10)}$$

The corresponding Fourier spectrum is the convolution of the spectrum of the cosine with peaks of amplitude $1/2$ at $\pm\omega_1$ with the spectrum of the pulse train. The two peaks of the cosine are shifted to the positions of all the peaks of the pulse train and multiplied with their amplitude. Always two neighboring peaks of the pulse train at odd multiples of ω_1 contribute to a peak at even multiples of ω_1 with amplitude

$$\tilde{a}_k = \frac{1}{2} c_{k+1} + \frac{1}{2} c_{k-1} = \frac{1}{\pi}(-1)^{\frac{k}{2}} \frac{1}{1-k^2}, \; k \text{ even} \quad \text{(S11)}$$

The zero-frequency peak of the pulse train gives rise to peaks at $\pm\omega_1$ with amplitude

$$\tilde{a}_{\pm 1} = \frac{1}{2} c_0 = \frac{1}{4} \quad \text{(S12)}$$

The spectrum of the thresholded superimposed cosine waves (Fig. A1 C) is composed of the spectrum of the pulse train convolved with the spectrum of the carrier cosine, Eqs. (S11) and (S12), and with the spectrum of the stimulus cosine with peaks of amplitude $\alpha / 2$ at frequencies $\pm\omega_2$. For the latter, each peak of the pulse train at odd multiples of ω_1 is replaced by a pair of peaks at frequencies $k\omega_1 \pm \omega_2$ with amplitudes

$$a_k = \frac{\alpha}{2} c_k = \frac{\alpha}{2} \frac{1}{\pi k} (-1)^{\frac{k-1}{2}}, \; k \text{ odd} \quad \text{(S13)}$$

These amplitudes are negative, i.e. they introduce a phase shift by π, for every second odd k ($k=3$, 7, 11, …).

The zero-frequency peak of the pulse train, Eq. (S9), gives rise to two peaks at $\pm\omega_2$ with amplitude

$$a_0 = \frac{\alpha}{2} c_0 = \frac{\alpha}{4} \quad \text{(S14)}$$

The relative amplitudes $\bar{a}_k = a_k / a_0$ up to $k=5$ multiples of ω_1 of the envelope frequencies introduced by thresholding are $\bar{a}_0 = 100$ %, $\bar{a}_1 = \frac{2}{\pi} \approx 64$ %, $\bar{a}_2 = 0$, $\bar{a}_3 \approx -\frac{2}{3\pi} = -21$ %, $\bar{a}_4 = 0$, and $\bar{a}_5 \approx \frac{2}{5\pi} = 13$ %.

4. Threshold cubed

Taking the signal, Eq. (1), to the power of three results in

$$x^3(t) = (\cos(\omega_1 t) + \alpha\cos(\omega_2 t))^3 \quad \text{(S15)}$$

$$\begin{aligned}
&= \frac{3}{4}(1+2\alpha^2)\cos(\omega_1 t) + \underline{\frac{3}{4}(2\alpha+\alpha^3)\cos(\omega_2 t)} \\
&\quad + \frac{1}{4}\cos(3\omega_1 t) + \frac{1}{4}\alpha\cos(3\omega_2 t) \\
&\quad + \underline{\frac{3}{4}\alpha\cos((2\omega_1+\omega_2)t)} + \underline{\frac{3}{4}\alpha\cos((2\omega_1-\omega_2)t)} \\
&\quad + \frac{3}{4}\alpha^2\cos((\omega_1+2\omega_2)t) + \frac{3}{4}\alpha^2\cos((\omega_1-2\omega_2)t)
\end{aligned} \quad \text{(S16)}$$

(Fig. A2 A). The dominant peaks depending on ω_2 are at ω_2 and $|2\omega_1 \pm \omega_2|$ (underlined).

Convolving the spectrum of the cubed signal, Eq. (S16), with the one of the pulse train, Eqs. (S8) and (S9), approximating the threshold operation, Eq. (2), boils down to replace all the peaks in the spectrum of the pulse train with the ones of the cubed signal shifted to the respective positions (Fig. 6 A–C). In the following calculations we ignore all terms of higher order in α.

The two purely ω_1-dependent terms with peaks at $\pm\omega_1$ and $\pm 3\omega_1$ result in peaks at even multiples of ω_1 with amplitudes

$$\begin{aligned}
\tilde{b}_k &= \frac{1}{2}\frac{3}{4}(c_{k+1}+c_{k-1}) + \frac{1}{2}\frac{1}{4}(c_{k+3}+c_{k-3}) \\
&= \frac{3}{4\pi}(-1)^{\frac{k}{2}}\left(\frac{1}{1-k^2} - \frac{1}{9-k^2}\right),\ k \text{ even}
\end{aligned} \quad \text{(S17)}$$

and in addition in peaks directly at $\pm\omega_1$ and $\pm 3\omega_1$ with amplitudes

$$\tilde{b}_{\pm 1} = \frac{1}{2}\frac{3}{4}c_0 = \frac{3}{16} \quad \text{(S18)}$$

$$\tilde{b}_{\pm 3} = \frac{1}{2}\frac{1}{4}c_0 = \frac{1}{16} \quad \text{(S19)}$$

(horizontal lines in Fig. A2 B). The latter at the third harmonics of ω_1 is a new peak that the threshold without exponent does not generate.

Convolving the dominant ω_2 dependent terms in Eq. (S15) with the peaks at odd multiples of ω_1 of the pulse train, Eq. (S8), we get peaks at $k\omega_1 \pm \omega_2$ for odd k with amplitudes

$$\begin{aligned} b_k &= \frac{1}{2}\frac{3}{4}2\alpha c_k + \frac{1}{2}\frac{3}{4}\alpha(c_{k+2} + c_{k-2}) \\ &= \frac{3}{\pi}\alpha(-1)^{\frac{k+1}{2}}\frac{1}{k(k^2-4)}, \quad k \text{ odd} \end{aligned} \quad \text{(S20)}$$

These are peaks at the same frequencies as for the threshold without exponent, but with different amplitudes.

However, from the convolution with the zero-frequency term of the pulse train, Eq. (S9), we get additional peaks at $\pm\omega_2$ and $\pm(2\omega_1 \pm \omega_2)$ with amplitudes

$$b_0 = \frac{1}{2}\frac{3}{4}2\alpha c_0 = \frac{3}{8}\alpha \quad \text{(S21)}$$

$$b_{\pm 2} = \frac{1}{2}\frac{3}{4}\alpha c_0 = \frac{3}{16}\alpha \quad \text{(S22)}$$

The latter is the one the power of three adds to the folding frequencies around the second multiple of ω_1 (Fig. A2 B).

The relative amplitudes $\bar{b}_k = b_k / b_0$ of the envelope frequencies introduced by a cubed threshold are all positive and read $\bar{b}_0 = 100$ %, $\bar{b}_1 = \frac{8}{3\pi} \approx 85$ %, $\bar{b}_2 = \frac{1}{2} = 50$ %, $\bar{b}_3 \approx \frac{8}{15\pi} = 17$ %, $\bar{b}_4 = 0$, and $\bar{b}_5 \approx \frac{8}{105\pi} = 2.4$ %.

5. Thresholding a SAM

A sinusoidal amplitude modulation (SAM) of frequency $\Delta\omega$ and amplitude α multiplies a carrier signal with frequency ω_1:

$$x(t) = (1+\alpha\cos(\Delta\omega t))\cos(\omega_1 t) \quad \text{(S23)}$$

According to the convolution theorem the spectrum of this signal is a convolution of the spectrum of the carrier with peaks at $\pm\omega_1$ and amplitude $\frac{1}{2}$ with the spectrum of the amplitude modulation with a peak of amplitude 1 at 0 and two peaks at $\pm\Delta\omega$ with amplitudes $\frac{\alpha}{2}$. The resulting spectrum of a SAM signal has peaks at $\pm\omega_1$ with amplitude $\frac{1}{2}$, at $\pm(\omega_1+\Delta\omega) = \pm\omega_2$ with amplitude $\frac{\alpha}{4}$, and at $\pm(\omega_1-\Delta\omega) = \pm(2\omega_1-\omega_2)$ also with amplitude $\frac{\alpha}{4}$. The latter are additional peaks that are not present in the superimposed cosine signal, Eq. (1).

For a SAM the threshold operation, Eq. (2), can be replaced by a multiplication with a pulse train, Eqs. (3) and (4), for all stimulus amplitudes $\alpha < 1$, because the amplitude modulation does not change the zero crossings of the signal. Using the results from above, the convolution of the peaks at $\pm\omega_1$ with the pulse spectrum results in peaks at even multiples of ω_1 and at ω_1 with amplitudes Eqs. (S11) and (S12), respectively. The convolution of the peaks at $\pm\omega_2$ results in peaks at $k\omega_1 \pm \omega_2$ for odd k and $k=0$ with half of the amplitudes given in Eqs. (S13) and (S14), respectively. The new peaks of the SAM at $\pm(\omega_1-\Delta\omega)$ get shifted to the peaks of the pulse train and appear at $k\omega_1 \pm (\omega_1-\Delta\omega) = (k\pm 2)\omega_1 \mp \omega_2$ for odd k with half the amplitudes of Eq. (S13) and for $k=0$ at $\pm(2\omega_1-\omega_2)$ with half the amplitude of Eq. (S14). These latter peaks fill in envelope frequencies close to two multiples of ω_1

6. Harmonics of the carrier

In reality the carrier EOD is a complex periodic wave and thus already provides harmonics at multiples of the carrier frequency. Wouldn't that be enough to explain the aliasing structure of the signal envelopes without non-linearities?

At least a threshold is needed. Without any non-linearity the only frequency component depending on the stimulus frequency still would be the stimulus itself. With a threshold, Eq. (2), approximated by multiplication with a pulse train, Eq. (4), the harmonics of the carrier EOD would only add peaks to the resulting spectrum at multiples of the carrier frequency. The stimulus frequency still would be just convolved with the spectrum of the pulse train. As for the sine-wave carrier, the stimulus frequency would appear as envelope frequencies around odd multiples of the carrier frequency and around zero frequency, Eqs. (S13) and (S14), but not at even multiples.

However, the harmonics of the carrier EOD modify the waveform. It is not a sine wave any more that stays positive for exactly half of the time and negative for the other half. Instead, the harmonics might distort the waveform such that we would need a pulse train with a duty cycle other than 50 % to emulate a threshold. For example, some *A. leptorhynchus* have a waveform that is wider than a sine wave at its zero crossings (Fig. 9 A). A matching pulse train would need a higher duty cycle (Fig. 9 B). We parameterize the pulse train by its duty cycle δ to account for this effect:

$$p(\omega_1 t;\delta) = \begin{cases} 1 & ; \quad -\delta\frac{\pi}{\omega_1} < t \bmod \frac{2\pi}{\omega_1} < \delta\frac{\pi}{\omega_1} \\ 0 & ; \quad \text{else} \end{cases} \quad \text{(S24)}$$

Changing the duty cycle modifies the spectrum of the pulse train:

$$c_k(\delta) = \frac{1}{\pi k}\sin(\pi k\delta),\ \ k \neq 0 \ \ \text{(S25)}$$

$$c_0(\delta) = \delta,\ \ k = 0 \ \ \text{(S26)}$$

(Fig. 9 B). In particular, a peak at the second multiple of the carrier appears with amplitude $c_2(\delta) = \frac{1}{2\pi}\sin(2\pi\delta)$. This peak is then convolved with the stimulus and fills in envelope frequencies around the second multiple (Fig. 9 C). The amplitude of the second multiple of the pulse train equals zero for $\delta = \frac{1}{2}$, grows linearly in δ according to $c_2(\delta) \approx \frac{1}{2} - \delta$ as the duty cycle deviates from $\frac{1}{2}$. It can get as large as the one of the fundamental, if, according to $c_2 / c_1 = \cos(\pi\delta)$, the duty cycle approaches zero or one. However, the envelope frequencies at the third multiple are missing now, because the third multiple of the pulse train is reduced by increasing the duty cycle.

To summarize, the harmonics of the carrier themselves do not contribute to extracting envelope frequencies. However, the changed duty cycle of the carrier waveform modifies the spectrum of the corresponding pulse train needed to approximate the threshold operation. Depending on the duty cycle of the carrier some harmonics are enhanced whereas others are suppressed.

7. Harmonics of the stimulus

Alternatively, we could keep the carrier as a sine wave and use a realistic EOD for the stimulus. The components of the signal spectrum relevant for explaining envelope frequencies result from the convolution of the spectrum of a pulse-train with a 50 % duty cycle, Eqs. (S8) and (S9), matching the sinusoidal carrier, with all the harmonics of the stimulus. For extracting the aliasing structure of the envelopes, however, only the fundamental of the stimulus is relevant. The higher harmonics introduce frequencies depending on multiples of the stimulus frequency and thus can not explain the envelope frequencies that grow directly proportionally with stimulus frequency.

8. Tuning of P-units to EOD frequency

Silencing the fish's EOD and measuring the minimum amplitude of an artificial replacement EOD to make a P-unit fire action potentials results in V-shaped threshold curves centered at the fish's EOD frequency (Fig. A3 A,[4]). The corresponding band-pass filter is probably caused by electric resonance in the electroreceptor cells[51]. This could be modeled by a damped harmonic oscillator filtering the input signal before it is thresholded at the receptor synapse[38].

To model this resonance filter we replaced the stimulus $x(t)$ in the P-unit models, Eqs. (A.1) – (A.4), by the output $y(t)$ of a harmonic oscillator

$$\frac{d^2y(t)}{dt^2} + 2\zeta w_0 \frac{dy(t)}{dt} + \zeta w_0^2 y(t) = x(t) \quad \text{(S27)}$$

multiplied with a normalization factor β. In Eq. (S27) the external force to the oscillator is the stimulus $x(t)$, ζ is the damping ratio of the harmonic oscillator, and

$$w_0 = \frac{w_R}{\sqrt{1-2\zeta^2}} \quad \text{(S28)}$$

is the eigenfrequency, where $w_R = 2\pi f_R$ is the resonance frequency that was set to the measured f_{EOD} of each fish. The normalization factor

$$\beta = 70 w_R \sqrt{(2w_0\zeta)^2 + (w_R^2 - w_0^2)^2 / w_R^2} \quad \text{(S29)}$$

ensures that the fish's EOD passes through the damped oscillator with a gain of one. The harmonic oscillator was solved using the differential equation solver from SciPy.

We varied ζ from 0.7 (almost no damping) to 0.1 (highest damping). A stronger damping factor of high frequencies can be compensated for by a higher exponent at the threshold, with several combinations yielding similar results.

A mild damping coefficient of $\zeta = 0.45$ and an exponent $p = 5$ were sufficient to reproduce both the tuning of P-unit responses to f_{EOD} as reported by Hopkins[4] (Fig. A3 B). This model still reproduces the responses to beats up to three multiples of the f_{EOD} (Fig. A3 C, D), suggesting that the EOD filter does not impede P-unit responses to high difference frequencies.

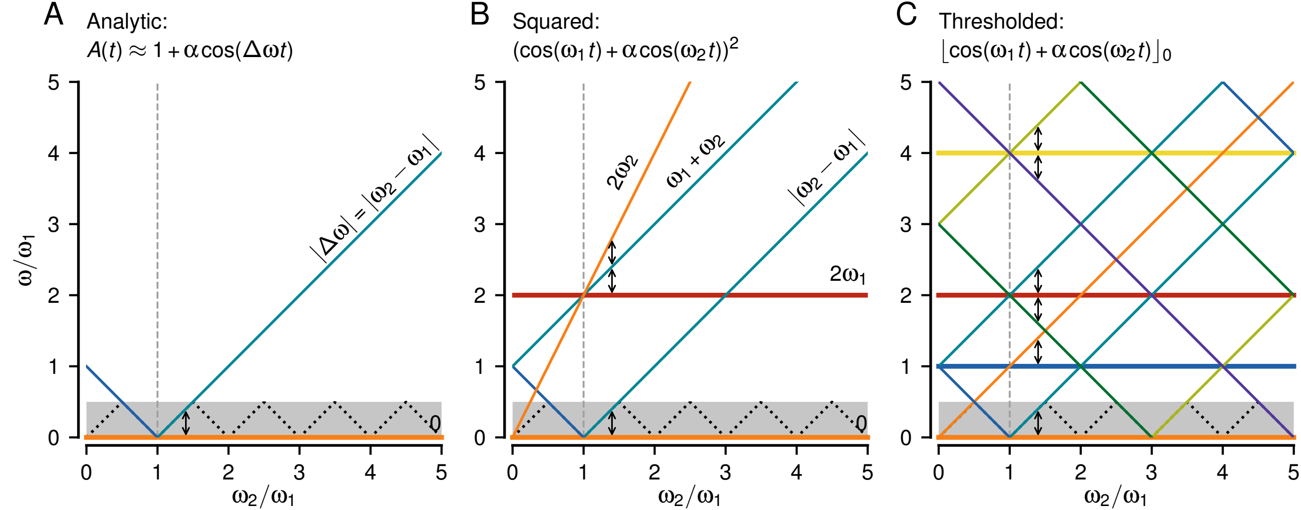

A
Analytic:
$A(t) \approx 1 + \alpha \cos(\Delta\omega t)$
$|\Delta\omega| = |\omega_2 - \omega_1|$
B
Squared:
$(\cos(\omega_1 t) + \alpha \cos(\omega_2 t))^2$
$2\omega_2$
$\omega_1 + \omega_2$
$|\omega_2 - \omega_1|$
$2\omega_1$
C
Thresholded:
$\lfloor \cos(\omega_1 t) + \alpha \cos(\omega_2 t) \rfloor_0$
ω/ω_1
ω_2/ω_1
0
1
2
3
4
5

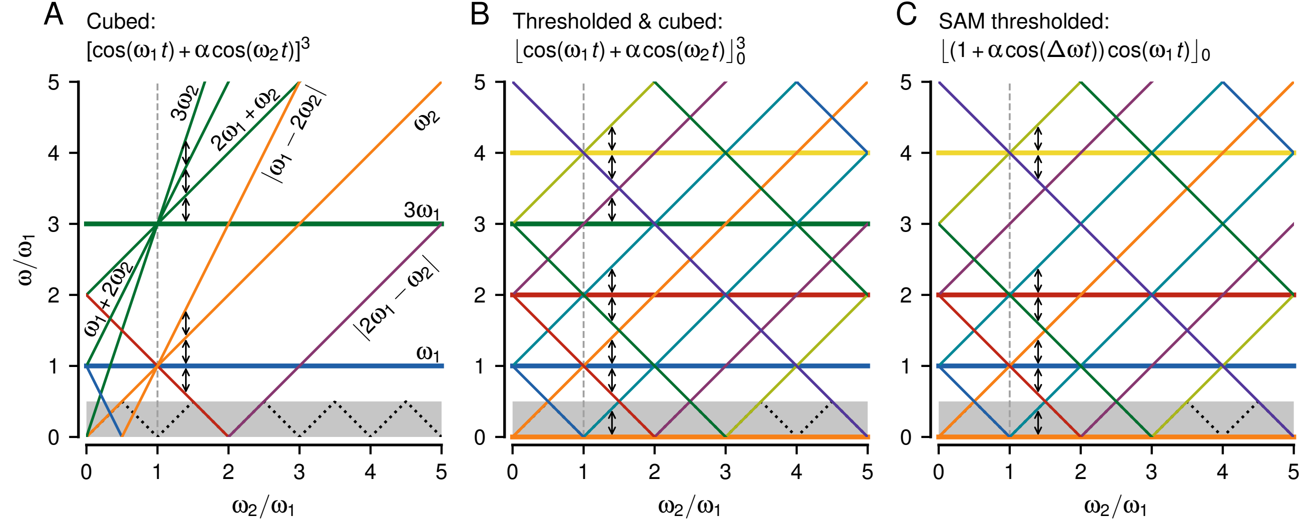

A
Cubed:
$[\cos(\omega_1 t) + \alpha\cos(\omega_2 t)]^3$
B
Thresholded & cubed:
$\lfloor \cos(\omega_1 t) + \alpha\cos(\omega_2 t) \rfloor_0^3$
C
SAM thresholded:
$\lfloor (1 + \alpha\cos(\Delta\omega t))\cos(\omega_1 t) \rfloor_0$
$3\omega_2$
$2\omega_1 + \omega_2$
$|\omega_1 - 2\omega_2|$
ω_2
$3\omega_1$
$\omega_1 + 2\omega_2$
$|2\omega_1 - \omega_2|$
ω_1
ω/ω_1
ω_2/ω_1
0
1
2
3
4
5

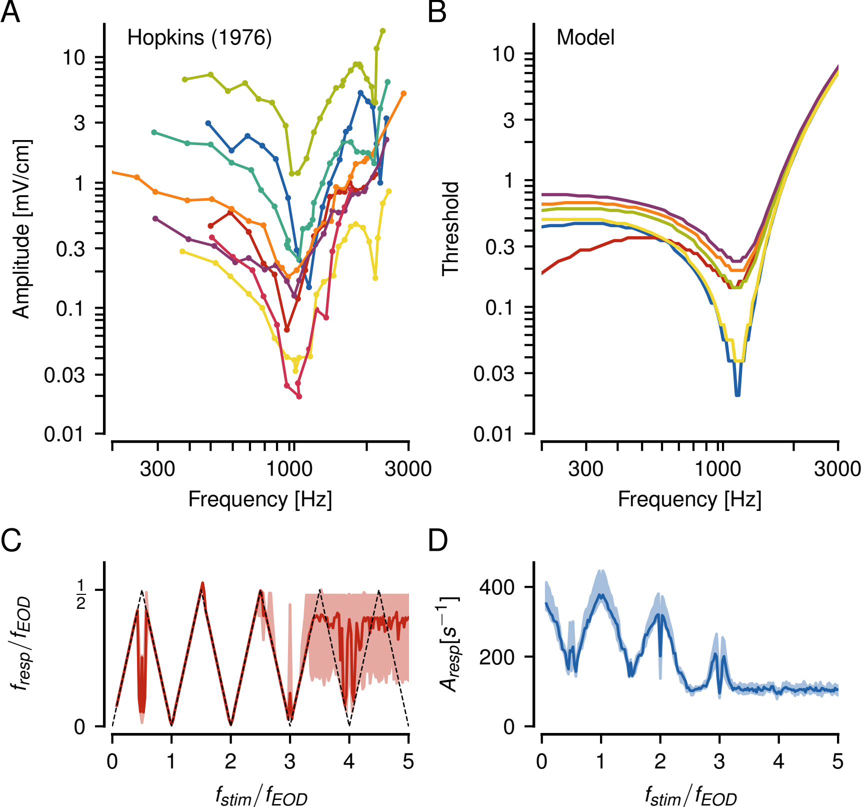

A
Hopkins (1976)
Amplitude [mV/cm]
10
3
1
0.3
0.1
0.03
0.01
300
1000
3000
Frequency [Hz]
B
Model
Threshold
10
3
1
0.3
0.1
0.03
0.01
300
1000
3000
Frequency [Hz]
C
f_{resp}/f_{EOD}
$\frac{1}{2}$
0
0
1
2
3
4
5
f_{stim}/f_{EOD}
D
$A_{resp}[s^{-1}]$
400
200
0
0
1
2
3
4
5
f_{stim}/f_{EOD}

Figure A1: Spectral peaks in analytic, squared and thresholded signals of superimposed cosine waves, Eq. (1). Related to Fig. 5. Plotted are the positions of peaks in the spectrum as a function of the stimulus frequency ω_2 relative to the carrier frequency ω_1. For a given stimulus frequency, the corresponding spectrum is a vertical slice through the graph. Vertical arrows highlight the difference frequency $\Delta\omega = \omega_2 - \omega_1$. Frequencies below $\omega_1 / 2$ are marked by the gray background at the bottom. The black dotted line in this frequency band indicates the folded frequencies at $\omega_f = | \omega_2 - \omega_1 \lfloor \omega_2 / \omega_1 \rceil |$. **A** The amplitude modulation Eq. (S5) computed as the magnitude of the analytic signal by means of a Hilbert transform has peaks only at 0 and at the absolute difference frequency $|\Delta\omega|$. **B** Squaring also generates the difference frequency and an offset at zero frequency. In addition, three more peaks appear at $2\omega_1$, $2\omega_2$, and $\omega_1 + \omega_2$, Eq. (S7). **C** Thresholding the signal results in many more peaks. Convolution of the spectrum of the pulsetrain, Eqs. (S8) and (S9), with the one of the carrier results in horizontal lines at even multiples of ω_1, Eq. (S11), and at ω_1, Eq. (S12). Of interest, however, are the peaks that depend on ω_2. They appear around odd multiples of ω_1, Eq. (S13), and around 0, Eq. (S14).

Figure A2: Spectral peaks in cubed and cubed-thresholded superimposed cosine waves as well as for thresholded SAMs. Related to Fig. 6. Same style as Fig. A1. **A** Spectral peaks, Eq. (S15), resulting from taking the signal to the power of three. **B** Major peaks resulting from thresholding and cubing the signal. Peaks not depending on ω_2 (horizontal lines) appear at even multiples of ω_1, Eq. (S17), at ω_1, Eq. (S18), and $3\omega_1$, Eq. (S19). The dominant stimulus-dependent peaks (diagonal lines) appear around odd multiples of ω_1, Eq. (S20), and the zeroth multiple, Eq. (S21), as for the threshold operation without exponent. In addition, however, we get a stimulus-dependent peak around twice the carrier frequency, Eq. (S22). **C** All spectral peaks of a thresholded SAM stimulus, Eq. (S23). Below half the carrier frequency (gray band) it results in the very same peaks as thresholded and cubed superimposed cosines. See supplement section Data S1 for details.

Figure A3: . Effects of the P-unit's EOD frequency filter. Related to STAR Methods. **A** Sensitivity of P-unit afferents to EOD frequency as reported by Hopkins[4]. The measured stimulus amplitudes were the minimum amplitude required to elicit a just noticeable difference in firing rates of the P-units. **B** Corresponding sensitivities of our LIF models supplemented by a harmonic oscillator Eq. (S27). The amplitudes elicited an increase in firing rate of 10 % compared to baseline rate without stimulus. **C & D** The frequency f_{resp} and corresponding response amplitudes of these P-unit models to beats still reproduce our observed P-unit tuning to beats despite the EOD filter.
